# Hydrolytically Stable and Cytotoxic [ONO*N*]_2_Ti(IV)-Type Octahedral Complexes

**DOI:** 10.1021/acs.inorgchem.2c02737

**Published:** 2022-10-23

**Authors:** Anastasia Pedko, Eden Rubanovich, Edit Y. Tshuva, Avital Shurki

**Affiliations:** † Institute of Chemistry, Edmond J Safra Campus, The Hebrew University of Jerusalem, Jerusalem9190401, Israel; ‡ Institute for Drug Research, School of Pharmacy, Ein Kerem Campus, The Hebrew University of Jerusalem, Jerusalem9112001, Israel

## Abstract

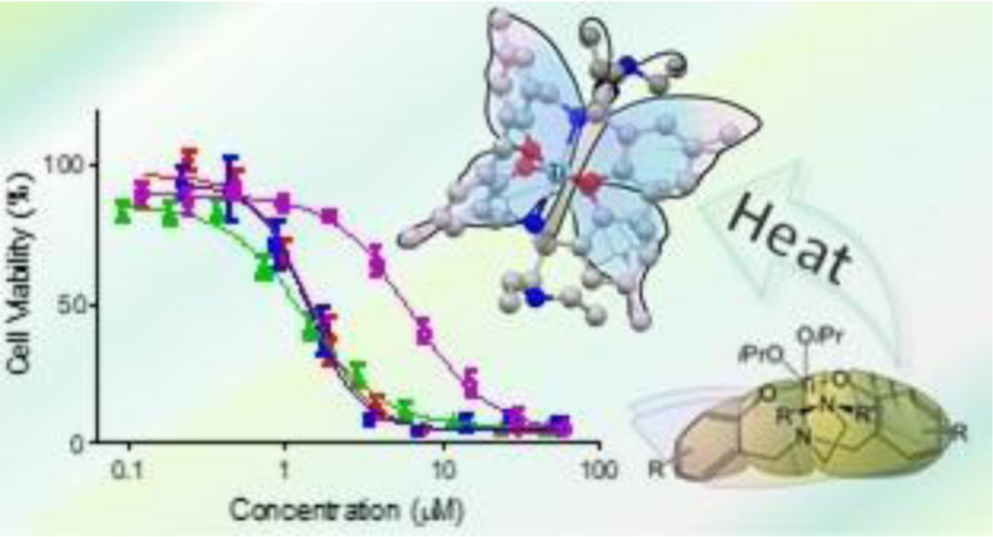

A new family of titanium(IV) complexes based on [ONON] diaminobis(phenolato) ligands with Me, Br, Cl, and F ortho substitutions was synthesized and characterized. X-ray structures of three derivatives revealed homoleptic L_2_Ti-type compounds that exhibit an octahedral geometry without binding of the dangling amine unit. DFT calculations demonstrated that the preference of an L_2_Ti complex is not driven by solvent or ligand substitutions but rather by entropic effects. Except for the fluorinated derivative that was hydrolyzed immediately following water addition at room temperature and had the lowest biological activity of the series tested, all other complexes showed cytotoxic activity comparable to or higher than (up to 10-fold) that of cisplatin toward human ovarian A2780 and colon HT-29 cancer cell lines (IC_50_ values: 0.6–13 μM after incubation for 72 h). Activity was generally higher (up to 10-fold) toward the more sensitive ovarian line and similar for all active complexes, whereas differences were recorded toward the colon line that are attributed to bioavailability variations among the complexes analyzed. Particularly high hydrolytic stability was recorded for the brominated derivative with a *t*_1/2_ of 17 ± 1 days for ligand hydrolysis in 10% D_2_O at room temperature, relative to *t*_1/2_ of 56 ± 5 and 22 ± 6 h measured for the chlorinated and methylated derivatives, respectively. Altogether this series of compounds represent a promising family of anticancer agents, with the chlorinated derivative showing the best combination of stability, cytotoxicity, and bioavailability.

## Abstract 2

A new series of homoleptic [ONO*N*]_2_Ti-type complexes with potential anticancer activity was synthesized, with the chlorinated derivative showing the best combination of stability, cytotoxicity, and bioavailability.

## Introduction

The disadvantages of cisplatin related to toxicity and resistance development prompt the quest toward alternative drugs based on other transition metals,^1−7^ among which is titanium(IV).^8−16^ Titanium(IV) coordination complexes are biocompatible; their final hydrolysis product, titanium dioxide, is known as a safe material, often present in food and cosmetic products, presenting an advantage for medicinal use.^17−19^ Titanocene dichloride and budotitane were the first Ti(IV) complexes analyzed for antitumor applications and reached clinical trials due to several attractive features: (a) they are effective in vivo;^11,13^ (b) they show reduced toxic effects in animals;^11,12^ and (c) they are active toward cells resistant to cisplatin.^11,12,20−22^ Nevertheless, the hydrolytic instability of these complexes eventually led to their failure in clinical trials as rapid formation of inactive aggregates was clinically ineffective.^14,23^

**Scheme 1:**
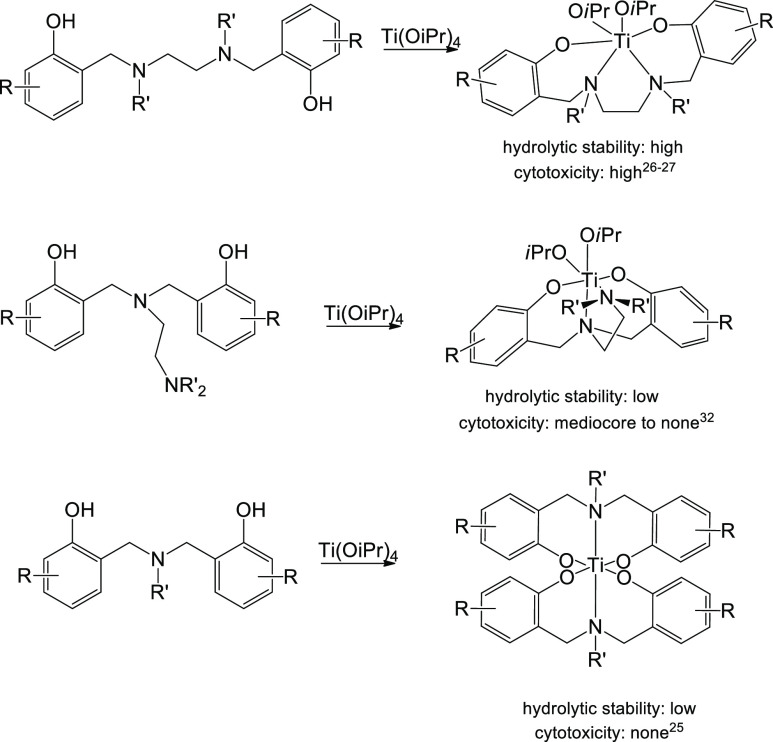
Aminephenolato Ligands of [ONNO] (Top), [ONON] (Middle), and [ONO] (Bottom) Type Give LTiX_2_-, LTiX_2_-, and L_2_Ti-Type Complexes upon Reaction with Ti(OiPr)_4_, Respectively; Hydrolytic Stability of Complexes: High *t*_1/2_ for Labile Ligand Hydrolysis in 10% D_2_O at Room Temperature > 5 h; Low *t*_1/2_ for All Ligand Hydrolysis at the Aforementioned Conditions < 10 min; Cytotoxicity of Complexes: High IC_50_ Values < 5 μM and Maximal Inhibition > 75%; Mediocre IC_50_ Values in the Range 5–50 μM and/or Maximal Inhibition < 75%; No IC_50_ Values > 50 μM and/or Maximal Inhibition < 20%

As a part of the quest toward advanced more stable and cytotoxic Ti(IV) complexes, our group has introduced amine phenolato complexes for antitumor applications.^24^ “Salan” complexes of [ONNO]-type diaminobis(phenolato) ligands gave highly cytotoxic complexes, with enhanced stability relative to classical titanocene or diketonato complexes. In these complexes, a single tetradentate ligand wraps around the metal to leave room for two additional labile ligands (Scheme 1, top).^25−31^ The *t*_1/2_ values for labile ligand hydrolysis were between a few hours for complexes with NMe donors and with alkylated aromatic rings to a few days for ortho-halogenated compounds, that is, ortho-brominated or -chlorinated derivatives.^27^ Interestingly, a complex with ethylated amine groups was highly unstable and consequently inactive.^26^ Another related family of aminobis(phenolato) complexes includes the [ONON]-type ligands with a dangling amine donor (Scheme 1, middle).^32−35^ These ligands also generally bind Ti(IV) through all four atoms, including the dangling amine,^33,34^ but weaker Ti–N bonds for the side arm lead to less stable and somewhat less active complexes. A ligand with ortho- and para-dimethylated aromatic rings and a diethylated side amine donor arm (Scheme 1, middle: R = Me, R′ = Et) is bound similarly to give an LTiX_2_-type complex, despite the relatively long Ti–N bond (2.46 Å) leading to negligible stability and activity.^32^ Homoleptic L_2_Ti complexes of bis(phenolato) ligands were observed only for tridentate [ONO]-type ligands (Scheme 1, bottom)^25,33,34^ and were mostly unstable and inactive, although some related complexes of mono-phenolato ligands exhibit stability and cytotoxicity.^25,36,37^

**Scheme 2:**
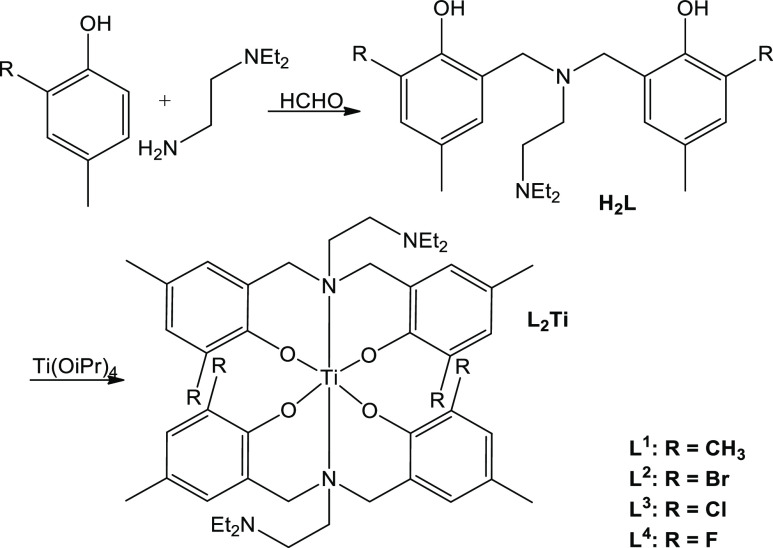
Synthesis of Diaminebis(phenolato) Ligands and Their Complexes

Herein, we report on octahedral [ONON]_2_Ti complexes, obtained from [ONON]-type ligands without binding of the side amine donor (Scheme 2). In particular, heating provided an entropic drive for second ligand binding without coordination of the dangling N donor. Additionally, ortho-methylation, -bromination, or -chlorination provide complexes of this family that are highly stable and highly cytotoxic toward human cancer cells.

## Experimental

### Materials and Physical Measurements

Starting materials 2-chloro-4-methylphenol, 2-fluoro-4-methylphenol (Chem Scene), 2-bromo-4-methylphenol, titanium(IV) isopropoxide (Aldrich), 2,4-dimethylphenol (Fluka), *N*,*N*-diethylethylenediamine (Alfa Aesar), formaldehyde 37% (J.T. Baker), and methanol (Bio-lab) were used as received. Tetrahydrofuran (Bio-lab) was dried by solvent purifiers MB-SPS. Elemental analysis of C, H, and N was performed on a Thermo Flash 2000 CHN-O elemental analyzer; Elemental analysis of Cl, Br, and F was performed by Anton Paar Microwave Induced Oxygen Combustion (MIC) for the decomposition of organic samples by Dionex LC20 ion chromatography. High-resolution mass spectrometry was conducted using a Q Exactive Plus mass spectrometer (Thermo Fisher Scientific) coupled with a Dionex UltiMate 3000 UPLC system (Thermo Fisher Scientific). Solutions for ^1^H and ^13^C NMR spectra were prepared in DMSO-*d*_6_ or THF-*d*_8_ (Cambridge Isotope Laboratories, INC), measured at 400 and 125 MHz, respectively, and analyzed with TopSpin 4.0.6, Bruker Corporation. The chemical shifts were referenced to Me_4_Si (δ 0.00 ppm) and DMSO-*d*_6_ (δ 2.5 ppm) or THF *d*_8_ (δ 3.58 and 1.72 ppm). Hydrolysis studies were performed by ^1^H NMR spectroscopy at 298 K with 32 scans using 1,4-dinitrobenzene (Sigma Aldrich) as the internal standard, recording the spectra at 500 MHz.

### Ligand Synthesis

Ligands were synthesized by mixing different ortho-substituted meta-methylphenols, *N*,*N*-diethylethylenediamine, and formaldehyde in a molar ratio of 2:1:2 in methanol under reflux for 24 h, as described previously.^34,38^ For H_2_L^4^, the reagents were mixed for a week under similar conditions, then the solvent was evaporated, and the product was recrystallized from methanol. The ligands were characterized by elemental analysis, ^1^H NMR and ^13^C NMR spectroscopy, and HRMS.

H_2_L^1^: Obtained as white powder in 45.76% (2.2 gr) yield. Anal. Found: C, 74.68; H, 9.49; N, 7.23. Calcd for C_24_*H*_36_*N*_2_*O*_2_: C, 74.96; H, 9.44; N, 7.28. ^1^H NMR (*C*_4_*D*_8_*O* 400 MHz) δ_H_: 8.99 (S, 2H, OH), 6.66 (d, *J* = 2 Hz, 2H, Ar–H), 6.55 (d, *J* = 2 Hz, 2H, Ar–H), 3.41 (S, 4H, *CH*_2_), 2.53 (t, *J* = 6 Hz, 2H, *CH*_2_), 2.44–2.34 (m, 6H, *CH*_2_), 2.04 (S, 6H, *CH*_3_), 2.00 (S, 6H, *CH*_3_), and 098 (t, *J* = 7 Hz, 6H, *CH*_3_) ppm. ^13^C NMR (*C*_4_*D*_8_*O* 125 MHz): 151.6, 132.4, 129.9, 128.7, 124.1, 110.3, 66.9, 54.9, 49.6, 49.1, 45.6, 19.1, and 9.6 ppm. HRMS: (C_24_H_36_N_2_O_2_ + H^+^) *m*/*z* Calcd: 385.28495. Found: 385.28497.

H_2_L^2^: Obtained as white powder in 65.75% (2.87 gr) yield. Anal. Found: C, 52.01; H, 5.97; N, 5.49; Br, 29.51. Calcd for C_22_*H*_30_Br_2_*N*_2_*O*_2_: C, 52.19; H, 5.97; N, 5.29; Br, 30.19. ^1^H NMR ((*CD*_3_)_2_*SO* 400 MHz) δ_H_: 10.5 (S, 2H, OH), 7.21 (d, *J* = 2 Hz, 2H, Ar–H), 6.89 (d, *J* = 2 Hz, 2H, Ar–H), 3.59 (S, 4H, *CH*_2_), 2.61 (t, *J* = 5 Hz, 2H, *CH*_2_), 2.44 (q, *J* = 8 Hz 4H, *CH*_2_), 2.14 (S, 6H, *CH*_3_), and 0.96 (t, *J* = 7 Hz, 6H, *CH*_3_) ppm. ^13^C NMR ((*CD*_3_)_2_*SO* 125 MHz): 151.5, 132.4, 130.7, 125.3, 110.5, 54.6, 49.2, 45.6, 40.6, 20.1, and 10.5 ppm. HRMS: (C_22_H_30_Br_2_N_2_O_2_+H^+^) *m/z* Calcd: 515.07263. Found: 515.07227.

H_2_L^3^: Obtained as white powder in 30.07% (1.08 gr) yield. Anal. Found: C, 62.42; H, 6.94; Cl, 17.18; N, 6.59. Calcd forC_22_*H*_30_*Cl*_2_*N*_2_*O*_2_: C, 62.12; H, 7.11; Cl, 16.67; and N, 6.59. ^1^H NMR ((*CD*_3_)_2_*SO* 400 MHz) δ_*H*_: 7.07 (d, *J* = 2 Hz, 2H, Ar–H), 6.8 (d, *J* = 2 Hz, 2H, Ar–H), 3.6 (S, 4H, *CH*_2_), 2.61 (t, *J* = 6 Hz, 2H, *CH*_2_), 2.52–2.51 (m, 2H, *CH*_2_), 2.43 (q, *J* = 7 Hz, 4H, *CH*_2_), 2.16 (S, 6H, *CH*_3_), and 096 (t, *J* = 7 Hz, 6H, *CH*_3_) ppm. ^13^C NMR ((*CD*_3_)_2_*SO* 125 MHz): 150.4, 129.9, 129.4, 128.7, 125.5, 120.3, 54.39, 49.43, 49.18, 45.7, 20.2, and 10.6 ppm. HRMS: (C_22_H_30_Cl_2_N_2_O_2_ + H^+^) *m/z* Calcd: 425.17571. Found: 425.17578.

H_2_L^4^: Obtained as white powder in 66.3% (1.2 gr) yield. Anal. Found: C, 67.10; H, 7.61; F, 9.13; N, 7.06. Calc. for C_22_*H*_30_*F*_2_*N*_2_*O*_2_: C, 67.32; H, 7.70; F, 9.68; N, 7.14. ^1^H NMR ((*CD*_3_)_2_*SO* 400 MHz) δ_H_: 10.12 (S, 1H, OH), 6.87 (d, *J* = 2 Hz, 1H, Ar–H), 6.84 (d, *J* = 2 Hz, *J* = 2, 1H, Ar–H), 6.71 (S, 2H, Ar–H), 3.57 (S, 4H, *CH*_2_), 2.56 (t, *J* = 7 Hz, 2H, *CH*_2_), 2.49–2.45 (m, 2H, *CH*_2_), 2.37 (q, *J* = 7 Hz, 4H, *CH*_2_), 2.16 (S, 6H, *CH*_3_), and 091 (t, *J* = 7 Hz, 6H, *CH*_3_) ppm. ^13^C NMR (*C*_4_*D*_8_*O* 125 MHz): 152.33, 150.42, 142.62–142.52 (d, *J* = 12), 127.49–127.44 (d, *J* = 7), 125.81–125.55 (dd, *J* = 3, *J* = 30), 115.27–115.13 (d, *J* = 18), 53.96–53.94(d, *J* = 2), 49.66–49.48 (d, *J* = 22), 45.79, 19.48–19.47 (d, *J* = 2), and 10.05 ppm. HRMS: (C_22_H_30_F_2_N_2_O_2_:+H^+^) *m*/*z* Calcd: 393.23481. Found: 393.23483.

### Complex Synthesis

Titanium(IV) complexes were synthesized by mixing titanium(IV) isopropoxide and the ligand precursor in a molar ratio of 1:2 in dry THF at 61 °C for 24 h in a Universal oven UF 30 under an inert environment. The solvent was evaporated, and a red powder was obtained. The complexes were characterized by elemental analysis, ^1^H NMR and ^13^C NMR spectroscopy, and HRMS.

#### Synthesis of L^1^_2_Ti

The compound was obtained as red powder in quantitative yield. Anal. Found: C, 70.41; H, 8.46; N, 6.78. Calcd for C_48_*H*_68_*N*_4_*O*_4_*Ti*: C, 70.92; H, 8.43; N, 6.89. ^1^H NMR (*C*_4_*D*_8_*O* 400 MHz) δ_H_: 6.85 (d, *J* = 2 Hz, 1H, Ar–H), 6.77 (d, *J* = 1 Hz, 1H, Ar–H), 6.68 (t, *J* = 3 Hz, 2H, Ar–H), 4.721 (d, *J* = 13 Hz, 1H, *CH*_2_), 4.51 (d, *J* = 12 Hz, 1H, *CH*_2_), 3.68 (q, *J* = 9 Hz, 2H, *CH*_2_), 2.86–2.78 (m, 1H, *CH*_2_), 2.73–2.65 (m, 1H, *CH*_2_), 2.62–2.60 (m, 2H, *CH*_2_), 2.42–2.34 (m, 2H, *CH*_2_), 2.10 (S, 3H, Ar-CH_3_), 2.07 (S, 3H, Ar-CH_3_), 2.06–2.04 (m, 2H, *CH*_2_), 1.98 (S, 3H, Ar-CH_3_), 1.4 (S, 3H, Ar-CH_3_), and 0.64 (t, *J* = 7 Hz, 6H, CH_3_) ppm. ^13^C NMR (*C*_4_*D*_8_*O*125 *MHz*): 160.6, 159.7, 130.9, 130.6, 127.4 (d, *J* = 4), 126.3, 126.1, 122.7, 122.26 (d, *J* = 2), 122.23, 59.1, 58.3, 55.4, 47.5, 46.4, 42.8, 19.7, 19.6, 16.3, 16.05, 11.8, and 0.38 ppm. HRMS: (C_48_H_68_N_4_O_4_Ti + H^+^) *m/z* Calcd: 813.47928. Found: 813.47931.

Crystal data for C_48_H_68_N_4_O_4_Ti (*M* =812.96 g/mol): triclinic, space group *P*1̅ (no. 2), *a* = 11.1327(2) Å, *b* = 12.8122(2) Å, *c* = 17.5751(3) Å, α = 72.901(2)°, β = 83.726(2)°, γ = 66.857(2)°, *V* = 2203.14(8) Å^3^, *Z* = 2, *T* = 149.99(10) K, μ(Mo Kα) = 0.242 mm^–1^, *D*_calc_ = 1.225 g/cm^3^, 37,018 reflections measured (4.23° ≤ 2θ ≤ 64.854°), 12960 unique (*R*_int_ = 0.0346, *R*_sigma_ = 0.0365) which were used in all calculations. The final *R*_1_ was 0.0426 (*I* > 2σ(*I*)) and w*R*_2_ was 0.1144 (all data).

#### Synthesis of L^2^_2_Ti

The compound was obtained as red powder in quantitative yield. Anal. Found: C, 49.58; H, 5.48; N, 5.06; Br, 29.69. Calc. for C_44_*H*_56_*Br*_4_*N*_4_*O*_4_*Ti*: C, 49.28; H, 5.26; N, 5.22; Br, 29.80. ^1^H NMR ((*CD*_3_)_2_*SO* 400 MHz) δ_H_: 7.31 (d, *J* = 2 Hz, 2H, Ar–H), 7.27 (S, 4H, Ar–H), 7.23 (d, *J* = 2 Hz, 2H, Ar–H), 4.71 (d, *J* = 13 Hz, 4H, *CH*_2_), 4.06 (d, *J* = 13 Hz, 2H, *CH*_2_), 3.99 (d, *J* = 13 Hz, 2H, *CH*_2_), 3.14–3.05 (m, 2H, *CH*_2_), 2.70–2.63 (m, 4H, *CH*_2_), 2.22 (d, *J* = 7 Hz, 12H, Ar-CH_3_), 2.18–2.12 (m, 10H, *CH*_2_), and 0.71 (t, *J* = 7 Hz, 12H, *CH*_3_) ppm. ^13^C NMR (*C*_4_*D*_8_*O* 125 MHz): 158.3, 156.9, 132.9, 132.2, 129.4, 129.1, 128.7, 128.4, 124.47, 124.44, 109.3, 108.6, 67.2, 58.7, 58.1, 47.2, 46.5, 42.8, 25.39, 19.3, 19.2, and 11.7 ppm. HRMS: (C_44_H_56_Br_4_N_4_O_4_Ti + H^+^ (*m/z* Calcd: 1073.05464. Found: 1073.05627.

Crystal data for C_53_H_80.5_Br_4_N_4_O_7.25_Ti (*M* = 1257.25 g/mol): monoclinic, space group *P*2_1_/*n* (no. 14), *a* = 13.4994(6) Å, *b* = 22.0291(7) Å, *c* = 19.9404(8) Å, β = 104.712(4)°, *V* = 5735.5(4) Å^3^, *Z* = 4, *T* = 149.99(10) K, μ(Mo Kα) = 2.987 mm^–1^, *D*_calc_ = 1.456 g/cm^3^, 40031 reflections measured (3.626° ≤ 2θ ≤ 52°), 11,148 unique (*R*_int_ = 0.0681, *R*_sigma_ = 0.0675), which were used in all calculations. The final *R*_1_ was 0.1001 (*I* > 2σ(*I*)) and w*R*_2_ was 0.2040 (all data).

#### Synthesis of L^3^_2_Ti

The compound was obtained as red powder in quantitative yield. Anal. Found: C, 58.72; H, 6.49; Cl, 15.09; N, 5.92. Calcd for C_44_*H*_56_*N*_4_*Cl*_4_*O*_4_*Ti*: C, 59.07; H, 6.31; Cl, 15.85; N, 6.26.^1^H NMR ((*CD*_3_)_2_*SO* 400 MHz) δ_H_: 7.20 (d, *J* = 2 Hz, 1H, Ar–H), 7.17 (d, *J* = 2 Hz, 1H, Ar–H), 7.15 (d, *J* = 2 Hz, 1H, Ar–H), 7.09 (d, *J* = 2 Hz, 1H, Ar–H), 4.57 (dd, *J* = 13, *J* = 7 Hz, 2H, CH_2_), 4.06 (d, *J* = 14 Hz, 1H, CH_2_), 3.98 (d, *J* = 14 Hz, 1H, CH_2_), 3.02–2.94 (m, 1H, CH_2_), 2.64 (t, *J* = 8 Hz, 2H, CH_2_), 2.57–2.52 (m, 1H, CH_2_), 2.22–2.20 (d, *J* = 6 Hz, 6H, Ar-CH_3_), 2.18–2.15 (m, 1H, CH_2_), 2.14–2.13 (q, *J* = 4 Hz, 2H, CH_2_), 2.11–2.08 (m, 1H, CH_2_), and 0.7 (t, *J* = 7 Hz, 6H, CH_3_) ppm. ^13^C NMR (*C*_4_*D*_8_*O* 125 MHz): 157.2, 156.0, 129.88, 129.37, 128.61, 128.31, 128.19, 127.97, 124.64, 124.56, 119.32, 118.73, 67.23, 58.57, 57.89, 47.17, 46.47, 42.83, 25.40, 19.44, 19.36, and 11.74 ppm. HRMS: (C_44_H_56_N_4_Cl_4_O_4_Ti + H^+^) *m/z* Calcd: 895.25784. Found: 895.25867.

Crystal data for C_44.8_H_54.9_Cl_4_N_4_O_4.5_S_0.4_Ti (*M* =923.95 g/mol): triclinic, space group *P*1̅ (no. 2), *a* = 14.9277(2) Å, *b* = 17.5396(2) Å, *c* = 19.8460(3) Å, α = 98.5360(10)°, β = 111.6610(10)°, γ = 91.5980(10)°, *V* = 4756.40(12) Å^3^, *Z* = 4, *T* = 199.9(4) K, μ(Mo Kα) = 0.467 mm^–1^, *D*_calc_ = 1.290 g/cm^3^, 78,070 reflections measured (4.324° ≤ 2θ ≤ 58°), 25,126 unique (*R*_int_ = 0.0397, *R*_sigma_ = 0.0504) which were used in all calculations. The final *R*_1_ was 0.0637 (*I* > 2σ(*I*)) and w*R*_2_ was 0.1749 (all data).

#### Synthesis of L^4^_2_Ti

The compound was obtained as red powder in quantitative yield. Anal. Found: C, 63.19; H, 6.91; F, 8.70; N, 6.56. Calcd for C_44_*H*_56_*N*_4_*F*_4_*O*_4_*Ti*: C, 63.76; H, 6.81; F, 9.17; N, 6.76 (measurement affected by hydrolytic instability). ^1^H NMR ((*CD*_3_)_2_*SO* 400 MHz) δ_H_: 6.99 (d, *J* = 9 Hz, 2H, Ar–H), 6.87 (d, *J* = 11 Hz, 2H, Ar–H), 4.44 (d, *J* = 13 Hz, 1H, CH_2_), 4.34 (d, *J* = 14 Hz, 1H, CH_2_), 4.01 (d, *J* = 14 Hz, 2H, CH_2_), 2.81–2.60 (m, 4H, CH_2_), 2.22 (S, 3H, Ar-CH_3_), 2.19 (S, 3H, Ar-CH_3_), 2.16 (q, *J* = 7 Hz, 4H, CH_2_), and 0.71 (t, *J* = 7 Hz, 6H, CH_3_) ppm. ^13^C NMR (*C*_4_*D*_8_*O* 125 MHz): 148.8, 148.3, 125.64, 124.5, 124.3, 115.91, 66.1, 58.06, 47.17, 47.06, 43.25, 19.59–19.62 (d), 19.33, and 11.59 ppm. HRMS: (C_44_H_56_N_4_F_4_O_4_Ti + H^+^) *m/z* Calcd: 829.37899. Found: 829.37817.

### X-ray Crystallography

Single crystals of L^1^_2_Ti for X-ray crystallography were obtained by slow evaporation of acetonitrile at 254 K; single crystals of L^2^_2_Ti were obtained by slow evaporation of isopropanol at 254 K; and single crystals of L^3^_2_Ti were obtained by slow evaporation of DMSO solution at 298 K. The crystals were analyzed using an XtaLAB Synergy-S, single source at offset/far, HyPix diffractometer. The crystal was kept at 150 K during data collection. The structure was solved with the Olex2 and SHELXT structure solution program using intrinsic phasing and refined with the SHELXL refinement package using least squares minimization.^39−41^

### Computational Details

**Scheme 3:**
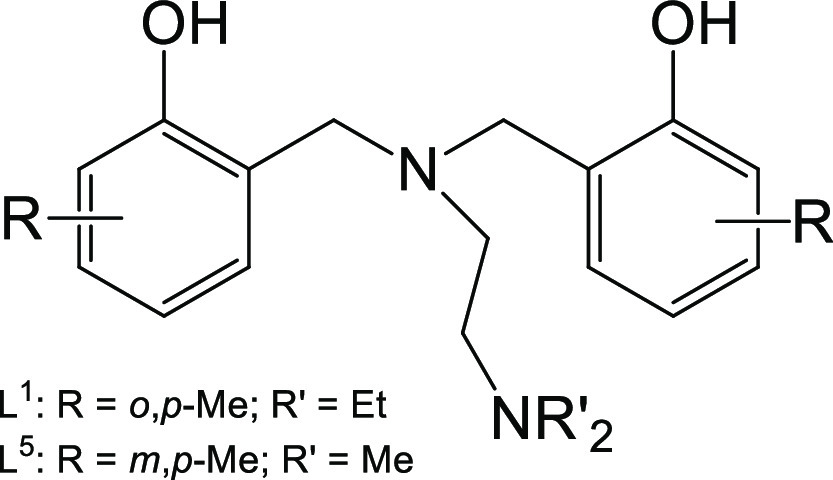
Ligands Employed for Analysis of Their Complexes in the Ligand-Exchange Reaction Presented in eq 1

Molecular geometries of the Ti bound meta- and para-methylated N-methylated^33^ (referred to here as structure L^5^Ti(OiPr)_2_ and the corresponding L^5^_2_Ti) and ortho- and para-methylated N-ethylated (structure L^1^Ti(OiPr)_2_ and the corresponding L^1^_2_Ti) complexes as well as that of the free ligands H_2_L^1,5^ (Scheme 3), and isopropanol ligands were optimized at the PBE0-D3 level of theory^42,43^ in *n*-pentane (ε = 1.8) and in acetonitrile (ε = 35.7) using the PCM solvation model.^44^ All calculations were performed using the Gaussian 16 software package^45^ employing Pulay’s m6-31G* basis set for the titanium atom^46^ and the 6-31G* basis set for the rest of the atoms.^47−49^ The PBE0 functional with a combination of basis sets was shown to provide a good compromise between the accuracy and computational cost for non-anionic transition-metal complexes.^50^ Optimizations were followed by frequency calculations to verify that the structures obtained are indeed minima along the potential energy surface and to determine thermochemical data (at 298.15 K and 1.0 atm). Optimized geometries of different calculated compounds as well as their absolute energies are available in the Supporting Information.

### Stability

Kinetic hydrolytic stability was examined by ^1^H NMR spectroscopy at 298 K with 32 scans. Ti(IV) complexes and 1,4-dinitrobenzene (Sigma Aldrich) as the internal standard were dissolved in DMSO-*d*_6_ or THF-*d*_8_ and 10% D_2_O was added as reported previously.^51^ The chemical shifts were calibrated to DMSO-*d*_6_ or THF-*d*_8_ (δ—2.5, δ—3.58 and 1.72 ppm, respectively). The integration of the peaks was analyzed with TopSpin 4.0.6, Bruker Corporation. To determine the half-life time, first the decay constant(*k*) was calculated from the slope of plotting the ln of the peak integral versus time (Figure S1) and then t_1/2_ was calculated as *t*_1/2_ = ln(2)/*k* (given as an average and STD of two to three repeats). All complexes are stable on the shelf in air as powder for at least 6 months.

### In Vitro Cytotoxicity

The cytotoxicity of Ti(IV) complexes was tested on A2780 human ovarian carcinoma cell line (European Collection of Authenticated Cell Cultures) and HT-29 human colorectal adenocarcinoma cell line (American Type Culture Collection). Cells were seeded in 96-well plates at 10,000 cells per well concentration, in complete RPMI 1640 medium, which contains 10% fetal bovine serum, 1% l-glutamine, and 1% penicillin/streptomycin (Biological Industries, Beit Haemek, Israel), and allowed to attach overnight. On the next day, a series of 10 different concentrations of the examined substances were prepared in either DMSO or THF: L^2,4^_2_Ti was diluted in DMSO and L^1,3^_2_Ti in THF. They were further diluted in growth medium and finally added to the cells, so that the final DMSO/THF concentration was 0.5%. Following a 3 day incubation at 37 °C in a 5% CO_2_ atmosphere, the cell viability was assessed by the 3-(4,5-dimethylthiazol-2-yl)-2,5-diphenyltetrazolium bromide (MTT) method.^52^ In a standard experiment, 20 μL of solution containing 0.1 mg of MTT (Sigma-Aldrich) was added to the cells and incubated for 3 h. The medium was replaced with 200 μL of isopropyl alcohol (Daejung), and the absorbance was measured at 550 nm using a Spark 10 M multimode microplate reader. Relative IC_50_ values were calculated by nonlinear regression of a variable slope model using GraphPad Prism 5.04 software ([top + bottom plateaus]/2). Error values were determined based on standard deviation. All measurements were repeated at least 3 × 3 times: 3 repeats per plate, all repeated three times on different days (nine repeats in total). Statistical validity (*p* < 0.1) was obtained for L^1,2^_2_Ti and L^1,4^_2_Ti for HT-29 and for L^2^_2_Ti for the two lines.

## Results and Discussion

### Structure

Ligands were synthesized by a Mannich condensation of substituted phenol, formaldehyde, and the diamine following procedures reported previously.^34,38^ The ^1^H NMR spectrum featured two aromatic peaks and new peaks at 3.6–3.41 ppm of four methylene protons. Elemental analysis, ^13^C NMR, and HRMS characterizations confirmed that the desired compounds had been obtained, with the expected symmetry manifested by the NMR results. The complexes were then synthesized by mixing the ligand and Ti(OiPr)_4_ in a molar ratio of 2:1 in dry THF at 61 °C for 24 h under an inert environment. The ^1^H NMR spectra confirmed the formation of L_2_Ti-type complexes (L^1–4^_2_Ti, Scheme 2), with shifts in the ligand peaks relative to the free ligand, and the lack of signals corresponding to bound isopropoxo groups. Such complexes have been previously obtained from related [ONO]-type tridentate ligands,^33,34^ whereas tetradentate [ONON] ones with an extra dangling amine donor have given the LTi(OiPr)_2_-type complexes with binding of both N atoms.^32−35^ Interestingly, applying a 1:1 ligand to metal ratio at room temperature did not lead to the formation of LTi(OiPr)_2_-type complexes as observed previously with similar salan ligands. The spectra each featured two sets of aromatic and aliphatic ligand signals, unlike the single set previously obtained for the salan LTi(OiPr)_2_-type ligands,^26,27^ except for the dangling arm presented by a single set of signals. These results imply that the two bound ligands are symmetrically related, whereas the ligand symmetry was abolished by different chemical environments for the two ligand sides. Two AB signal systems at ∼4.0–5.0 ppm for the methylene benzylic units reflect the constrained geometry of the bound complex.^33,34^^13^C NMR exhibited similar features, and HRMS and elemental analyses supported the L_2_Ti-type complex formation. Nevertheless, it could not be unequivocally determined based on the NMR alone whether the dangling amine group was bound to the metal center, although no constrained geometry was observed for the related signals.

**Figure 1:**
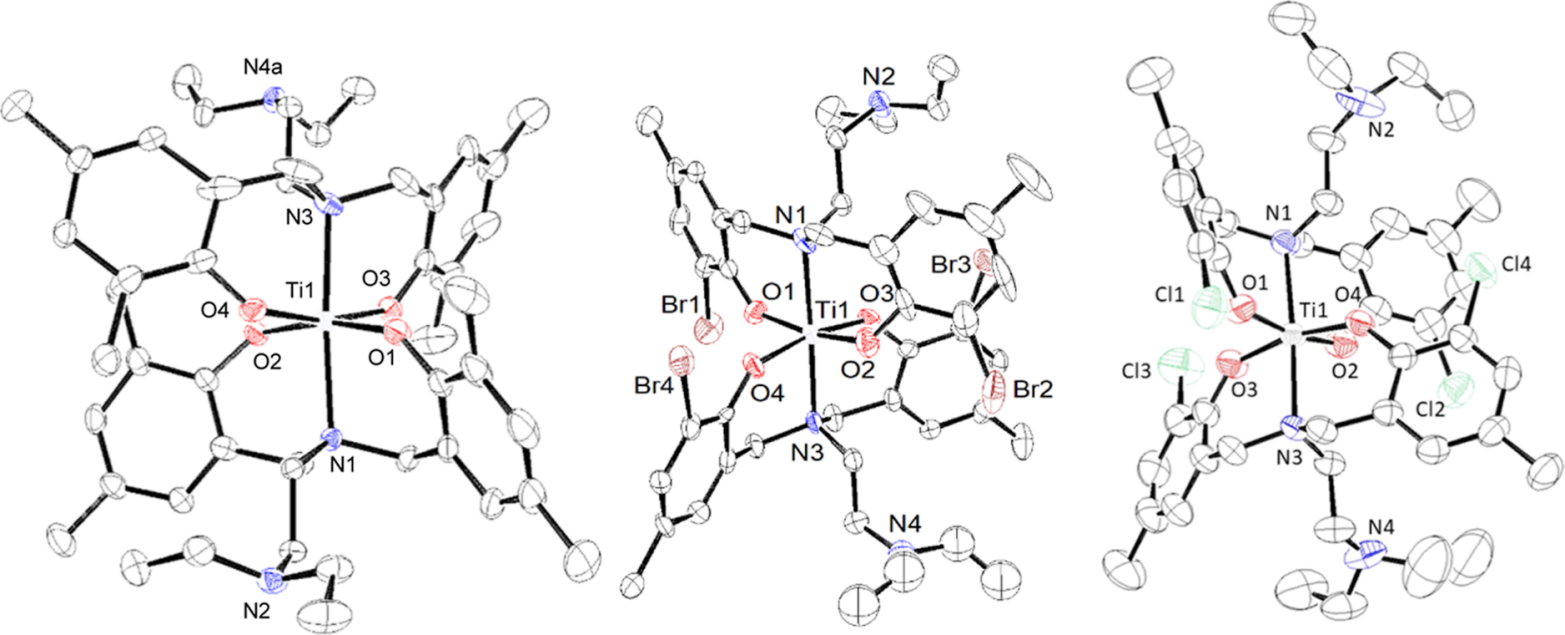
ORTEP drawing of L^1^_2_Ti (left), L^2^_2_Ti (middle), and L^3^_2_Ti (right) a 50% probability ellipsoids. Disorder in diethylamino groups was omitted for clarity.

Single crystals suitable for X-ray crystallographic analyses were obtained from slow evaporation of a cold acetonitrile solution of L^1^_2_Ti, a cold isopropanol solution of L^2^_2_Ti, and a DMSO solution of L^3^_2_Ti. Figure 1 depicts the ORTEP structure at 50% probability ellipsoids, and Table 1 summarizes selected bond lengths and angles.

**Table 1:** Selected Bond Lengths (Å) and Angles (°) for L^2^_2_Ti

	angle (deg)				bond distances (Å)		
atom	L^1^_2_Ti	L^2^_2_Ti	L^3^_2_Ti	atom	L^1^_2_Ti	L^2^_2_Ti	L^3^_2_Ti
O1–Ti1–O2	166.27(4)	169.2(3)	168.38(7)	Ti–O1	1.8795(9)	1.885(6)	1.895(2)
O1–Ti1–O4	89.97(4)	91.4(3)	92.41(8)	Ti–O2	1.8970(9)	1.885(6)	1.889(2)
O1–Ti1–N1	81.75(4)	84.1(3)	83.99(8)	Ti–O3	1.8764(9)	1.882(6)	1.879(2)
O1–Ti1–N3	95.47(4)	92.0(3)	91.73(7)	Ti–O4	1.8990(9)	1.873(6)	1.870(2)
O2–Ti1–O4	89.63(4)	89.6(3)	89.39(8)	Ti–N1	2.264(1)	2.254(8)	2.267(2)
O2–Ti1–N1	84.72(4)	85.1(3)	84.41(8)	Ti–N3	2.262(1)	2.245(8)	2.248(2)
O2–Ti1–N3	98.16(4)	98.8(3)	99.87(7)	Ti···N2	5.55	5.64	5.52
O1–Ti1–O3	94.05(4)	92.2(3)	91.75(8)	Ti···N4a	5.56	5.51	5.43
O2–Ti1–O3	89.56(4)	88.9(3)	89.03(9)				
O3–Ti1–O4	166.19(4)	168.3(3)	166.94(7)				
O3–Ti1–N1	95.83(4)	94.2(3)	96.25(7)				
O3–Ti1–N3	81.72(4)	84.7(3)	83.09(7)				
O4–Ti1–N1	97.82(4)	97.3(3)	96.49(7)				
O4–Ti1–N3	84.75(4)	84.0(3)	84.42(7)				
N1–Ti1–N3	176.18(4)	175.9(3)	175.65(8)				Pl

The structures of all three complexes are generally similar, featuring an octahedral metal center bound to two tridentate [ONO] ligands, supporting the NMR results with a solution symmetry of *C*_2_.^33^ The dangling amine groups did not bind the metal center, with >5 Å Ti–N distance and some disorder in the diethylamino groups. The structures evince that the two halves of each ligand are in different chemical environments, whereas both bound ligands are symmetrically related. The bond lengths and angles (Table 1) are like those found in related structures, indicative of strong covalent Ti–O and coordinative Ti–N bonds. As expected, the Ti–N bonds are similar to those previously reported for binding of the central amine and not to those found for the dangling amine in LTi(OiPr)_2_-type complexes, which are typically longer and indicative of weaker binding.^33,36^

Comparing the binding motif to that observed previously for [ONON]-type diaminobis(phenolato) ligands, it is interesting to note that homoleptic L_2_Ti complexes were obtained herein and not LTi(OiPr)_2_-type complexes as obtained previously.^33−35^ In particular, the exact same ligand with identical substitutions has previously produced an LTi(OiPr)_2_-type complex as analyzed crystallographically.^32^ One main difference in the reaction conditions stands out; the higher temperature applied herein, 61 °C versus room temperature, favoring the formation of the thermodynamic product. An additional difference involves the crystallization solvent: acetonitrile in the current study versus *n*-pentane in previous work.^32,33^ To determine whether the temperature is indeed the dominant factor in dictating the binding scheme, the change in enthalpy (Δ*H*) and the change in free energy (Δ*G*) were calculated at 298 K for the following ligand-exchange reaction in different solvents
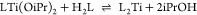
1Here, H_2_L is the free ligand. As various other similar salan complexes analyzed previously with different aromatic rings and N donor substituents gave similar LTi(OiPr)_2_-type complexes, similar calculations were conducted for a representative derivative bearing *meta*, *para-,* and *N*-methylation L^5^ (Scheme 3). The results of these calculations for both ligands are summarized in Table 2.

**Table 2:** Calculated Enthalpy and Free Energy (in kcal/mol) for Complexes in the Ligand-Exchange Reaction (eq 1) at 298.15 K and 1.00 atm in Different Solvents

	acetonitrile		*n*-pentane	
ligand	Δ*H*	Δ*G*	Δ*H*	Δ*G*
L^1^	11.4	–1.7	12.6	–0.5
L^5^	11.1	–2.3	12.5	–0.7

The differences between the calculated results for the two ligands as well as for the two solvents are very small, suggesting that the different structural aspects of the ligand as well as the different solvents did not dominate the resulting complex. The table also shows that enthalpy alone clearly favors the LTi(OiPr)_2_ over the L_2_Ti complexes for both ligands and in both solvents. Nevertheless, this clear preference disappears when free energy is considered, and the differences become virtually zero (with the L_2_Ti slightly favored in both cases). The large differences between the Δ*H* and the Δ*G* values suggest that the considered ligand-exchange reaction is entropy-driven, which indicates that heating is the main reason for obtaining the homoleptic L_2_Ti complexes in the current study.

### Hydrolysis

Hydrolysis studies were performed by adding 10% D_2_O (>100 equiv) to the complexes at room temperature to afford a pseudo-first-order reaction and monitoring the signal change in the ^1^H NMR spectra (Table 3). These conditions do not presume to reflect the biological environment but rather serve as a comparable tool to assess the stability relative to that of compounds analyzed under similar conditions as previously reported.^24−27,30−32^ For L^1–^^3^_2_Ti, decay in the integration of the complex signals was followed, ultimately indicating free ligand release. The methylated _2_Ti complex exhibited a high stability with *t*_1/2_ of ca. 1 day, which is markedly higher than that reported for [ONNO]TiX_2_-type alkylated complexes analyzed under similar conditions (*t*_1/2_ of several hours).^26,27^ The two halogenated complexes, L^2,3^_2_Ti, demonstrated an even higher stability with *t*_1/2_ values of several days, with the highest t_1/2_ recorded for the brominated derivative, L^2^_2_Ti, of more than 2 weeks. These results are consistent with previous observations that ortho-bromination or -chlorination enhances the hydrolytic stability of phenolato-Ti(IV) complexes, which may be attributed to the combination of steric and electronic influences.^27,32^ Steric hindrance may pose a kinetic barrier to the approach of water molecules, whereby additional electronic influences are provided by halogenation. Interestingly, the fluorinated derivative L^4^_2_Ti demonstrated low stability with instantaneous decomposition upon water addition (Figure S2), further emphasizing the importance of the steric parameter, with the potential contribution of H-bonding with water molecules in further reducing hydrolytic stability.

**Table 3:** *T*_1/2_ Values for Ligand Hydrolysis Measured for L^1–4^_2_Ti at Room Temperature following the Addition of >100 Water Equivalents

complex	ortho substitution	*t*_1/2_
L^1^_2_Ti	Me	22 ± 6 h
L^2^_2_Ti	Br	17 ± 1 days
L^3^_2_Ti	Cl	56 ± 5 h
L^4^_2_Ti	F	—a

a Decomposes spontaneously.

### Cytotoxicity

**Figure 2:**
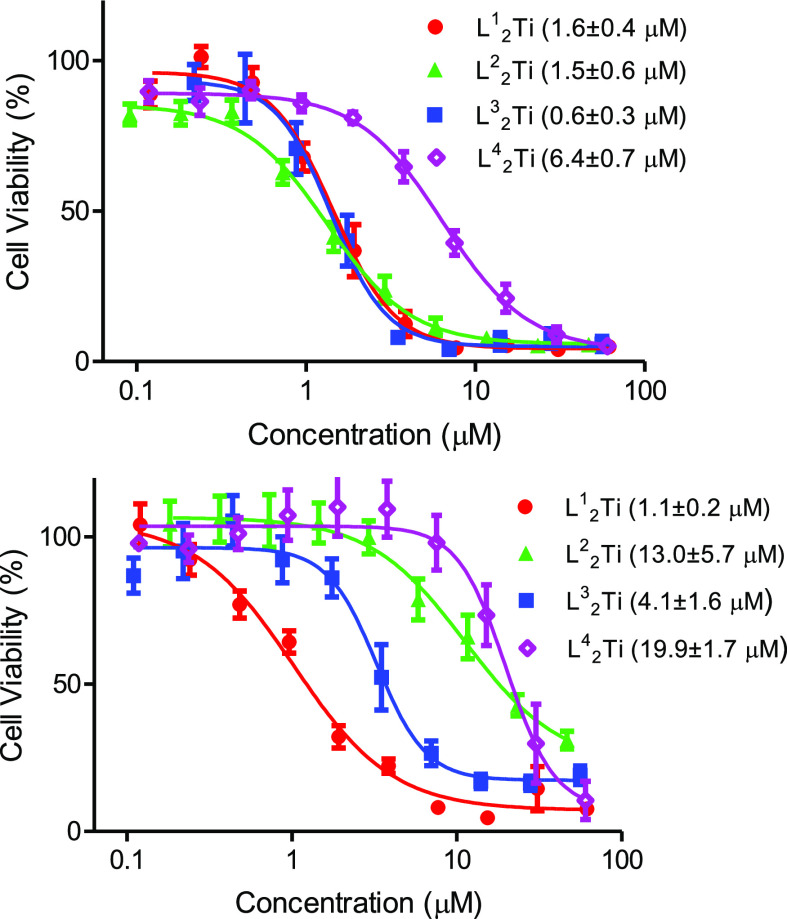
Dependence of human ovarian A2780 (top) and colon HT-29 (bottom) cancer cell viability on different concentrations (shown on a logarithmic scale) of L^1–4^_2_Ti, following a 3 day incubation period as analyzed by the MTT assay. Cisplatin IC_50_ = 1.6 ± 0.4 μM for A2780 and IC_50_ = 25 ± 4 μM for HT-29. H_2_L^1–4^ = 1.9 ± 0.7, 1.5 ± 0.1, 1.4 ± 0.6, 7.4 ± 1.5 μM for A2780 and IC_50_ = 1.3 ± 0.4, 10 ± 3, 10 ± 3, 26.9 ± 6.2 μM for HT-29 (see maximal inhibition values in Table S1). Relative IC_50_ values were calculated by nonlinear regression of a variable slope model by GraphPad Prism 5.04 software ([top + bottom plateaus]/2).

The cytotoxic activities of the complexes were tested on two types of cancer cell lines, A2780, human ovarian carcinoma cells and HT-29, human colorectal adenocarcinoma cells, and analyzed by the MTT assay.^52^Figure 2 depicts the dose–response curves of L^1–4^_2_Ti. The IC_50_ values are summarized in Figure 2. The complexes were highly active against both cell lines with activity that is comparable to, or greater than, that of cisplatin. Activity was also recorded for free ligands (Figure S3); nevertheless, previous studies indicated that any activity of ligands is unrelated to that of the complexes, as even complexes that were unstable and rapidly released the free ligands were inactive despite the activity of the free ligand itself, supporting the notion that the activity does not result from dissociated ligands.^26,27^ Ligand release is in any case of reduced relevance for the stable derivatives, L^1–3^_2_Ti, as similarly stable complexes have also been previously detected in their intact form in the cellular environment.^53^

Comparing the activity among the complexes reveals that all but the fluorinated derivative show similarly high activity toward the more sensitive ovarian line; nevertheless, differences were recorded on the more resistant colon line. Inspecting the series L^1–3^_2_Ti, activity is in opposite correlation to stability: the brominated complex is the least active despite its highest stability, which may be attributed to its large steric bulk, possibly affecting the bioavailability.^26,30,31,54^ Accordingly, the more lipophilic methylated derivative shows the highest activity of this series. The differences between the two lines may reflect different membrane permeability, affecting the insertion of bulkier, less bioavailable derivatives. As expected, L^4^_2_Ti, decomposing rapidly in water, showed markedly reduced cytotoxicity, presumably due to the instantaneous formation of less active or less bioavailable hydrolysis products.^26,27,30^

## Conclusions

A new series of homoleptic [ONO*N*]_2_Ti-type complexes is presented, which did not include the extra donor binding and yet showed high anticancer activity. Interestingly, for most derivatives, the high activity is combined with exceptional hydrolytic stability when compared with analogous derivatives.^24−27,32^ In previously reported work, the homoleptic [ONO]_2_Ti complex with no ortho substitutions was unstable and inactive,^25^ as well as most of the [ONON]TiX_2_ derivatives that included binding of the dangling amine;^32^ particularly, the derivative with ortho-methylated aromatic rings and ethylated dangling amine gave at room temperature an [ONON]TiX_2_-type complex that was unstable and inactive. In contrast, in the work presented here for ortho-substituted derivatives, upon heating, the dangling donor did not bind the metal and homoleptic [ONO]_*2*_Ti complex driven by entropic effects were obtained as supported by the calculations and still mostly provided high stability; in fact, by tuning complexation conditions, the exact same ligand with ortho-methylated aromatic rings and ethylated dangling amine gave an [ONO]_2_Ti-type complex of high stability and high activity. Other derivatives with ortho-halogenations produced compounds that are even more stable and markedly cytotoxic, mostly more than the clinically employed cisplatin.

The hydrolytic stability of the complexes is obviously valuable, as often unstable complexes decompose rapidly into inactive or non-bioavailable species and are therefore inactive.^26,30,32^ Nevertheless, the fair cytotoxicity of the fluorinated unstable complex should be attributed to its hydrolysis products (free ligand or clusters as observed in previous cases for relatively small unstable derivatives).^30,54,55^ Published work suggests that hydrolyzed free ligands of related compounds, if active, are not the source of activity even of relatively unstable complexes, probably due to the formation of ligand-bound inactive clusters.^26,27^ Nevertheless, the activity of H_2_L^4^ implies that its contribution to the activity of L^4^_2_Ti cannon be ruled out. For the stable derivatives L^1–3^_2_Ti, ligand hydrolysis is of less relevance as previous work has detected similarly stable derivatives in their intact form in the cell.^53^ For these derivatives, the cellular penetration seems to be a parameter of influence, especially as active transportation of some Ti(IV) cytotoxic compounds has been suggested:^55^ The cellular penetration may be affected by large steric bulk and general hydrophobicity/lipophilicity of the compounds and thus, along with solubility issues, may explain the activity pattern observed. As similarly hydrolytically stable derivatives have been observed in their intact form for days in the cellular environment,^55^ stable active complexes are good candidates to be employed for mechanistic investigations and eventually serve as potential drugs.

Altogether, considering all aspects, the chlorinated complex is identified as the derivative featuring the best combination of cytotoxicity, hydrolytic stability, and bioavailability of the series presented herein. We are currently looking into mechanistic aspects of these compounds and whether they may be correlated to other promising Ti(IV)-based anticancer drugs.

## Supporting Information Available

X-ray structures of L^1^_2_Ti (CCDC 2123949), L^2^_2_Ti(CCDC 2162882), and L^3^_2_Ti (CCDC 2168724); calculated geometries and absolute energies of different compounds; hydrolysis measurements; and cytotoxicity of free ligands (PDF)

## References

[ref1] Ayipo Y. O.; Adesina W.; Nizam M. Metal Complexes of β-Carboline: Advances in Anticancer Therapeutics. Coord. Chem. Rev. 2021, 432, 213746 10.1016/j.ccr.2020.213746.

[ref2] Aureliano M.; Gumerova N. I.; Sciortino G.; Garribba E.; Rompel A.; Crans D. C. Polyoxovanadates with Emerging Biomedical Activities. Coord. Chem. Rev. 2021, 447, 214143 10.1016/j.ccr.2021.214143.

[ref3] Paprocka R.; Wiese-Szadkowska M.; Janciauskiene S.; Kosmalski T.; Kulik M.; Helmin-Basa A. Latest Developments in Metal Complexes as Anticancer Agents. Coord. Chem. Rev. 2022, 452, 214307 10.1016/j.ccr.2021.214307.

[ref4] Bijelic A.; Aureliano M.; Rompel A. Polyoxometalates as Potential Next-Generation Metallodrugs in the Combat Against Cancer. Angew. Chem., Int. Ed. Engl. 2019, 58, 2980–2999. 10.1002/anie.201803868. 29893459 PMC6391951

[ref5] Peña Q.; Wang S.; Zaremba T.; Shi Y.; Scheeren H. W.; Metselaar J. M.; Kiessling F.; Pallares R. M.; Wuttke S.; Lammers T. Metallodrugs in cancer nanomedicine. Chem. Soc. Rev. 2022, 51, 2544–2582. 10.1039/d1cs00468a. 35262108

[ref6] Gourdon L.; Cariou G.; Gasser U.; Cariou K. Phototherapeutic anticancer strategies with first-row transition metal complexes: a critical review. Chem. Soc. Rev. 2022, 51, 1167–1195. 10.1039/d1cs00609f. 35048929

[ref7] Amante C.; Lu A. Vanadium and Melanoma : A Systematic Review. Metal 2021, 11, 828 10.3390/met11050828.

[ref8] Rafique S.; Idrees M.; Nasim A.; Akbar H.; Athar A. Transition Metal Complexes as Potential Therapeutic Agents. Biotechnol. Mol. Biol. Rev. 2010, 5, 38–45.

[ref9] Koepf-Maier P.; Koepf H. Non-Platinum Group Metal Antitumor Agents. History, Current Status, and Perspectives. Chem. Rev. 1987, 87, 1137–1152. 10.1021/cr00081a012.

[ref10] Meléndez E. Titanium Complexes in Cancer Treatment. Crit. Rev. Oncol. Hematol. 2002, 42, 309–315. 10.1016/s1040-8428(01)00224-4. 12050022

[ref11] Heim M. E.; Flechtner H.; Keppler B. K.. Clinical Studies with Budotitane—a New Non-Platinum Metal Complex for Cancer Therapy. In Ruthenium and Other Non-Platinum Metal Complexes in Cancer Chemotherapy; Springer, 1989; pp 217–223.

[ref12] Schilling T.; Keppler K. B.; Heim M. E.; Niebch G.; Dietzfelbinger H.; Rastetter J.; Hanauske A.-R. Clinical Phase I and Pharmacokinetic Trial of the New Titanium Complex Budotitane. Invest. New Drugs 1995, 13, 327–332. 10.1007/bf00873139. 8824351

[ref13] Keppler B. K.; Friesen C.; Moritz H. G.; Vongerichten H.; Vogel E. Tumor-Inhibiting Bis (β-Diketonato) Metal Complexes. Budotitane, Cis-Diethoxybis (1-Phenylbutane-1, 3-Dionato) Titanium (IV). Bioinorg. Chem. 1991, 97–127. 10.1007/3-540-54261-2_2.

[ref14] Toney J. H.; Marks T. J. Hydrolysis Chemistry of the Metallocene Dichlorides M (Η5-C5H5) 2C12, M= Ti, V, Zr. Aqueous Kinetics, Equilibria, and Mechanistic Implications for a New Class of Antitumor Agents. J. Am. Chem. Soc. 1985, 107, 947–953. 10.1021/ja00290a033.

[ref15] Ott I.; Gust R. Non Platinum Metal Complexes as Anti-cancer Drugs. Arch. Pharm. Int. J. Pharm. Med. Chem. 2007, 340, 117–126. 10.1002/ardp.200600151. 17315259

[ref16] Caruso F.; Rossi M.; Pettinari C. Anticancer Titanium Agents. Expert Opin. Ther. Pat. 2001, 11, 969–979. 10.1517/13543776.11.6.969.

[ref17] Tshuva E. Y.; Miller M. 8. Coordination Complexes of Ti(IV) for Anticancer Therapy. Metallo-Drugs: Development and Action of Anticancer Agents 2018, 219–250. 10.1515/9783110470734-008.

[ref18] Musial J.; Krakowiak R.; Mlynarczyk D. T.; Goslinski T.; Stanisz B. J. Titanium Dioxide Nanoparticles in Food and Personal Care Products—What Do We Know about Their Safety?. Nanomaterials 2020, 10, 1110 10.3390/nano10061110. PMC7353154 32512703

[ref19] Buettner K. M.; Valentine A. M. Bioinorganic Chemistry of Titanium. Chem. Rev. 2012, 112, 1863–1881. 10.1021/cr1002886. 22074443

[ref20] Köpf H.; Köpf-Maier P. Titanocene Dichloride—the First Metallocene with Cancerostatic Activity. Angew. Chem., Int. Ed. Engl. 1979, 18, 477–478. 10.1002/anie.197904771. 111586

[ref21] Lümmen G.; Sperling H.; Luboldt H.; Otto T.; Rübben H. Phase II Trial of Titanocene Dichloride in Advanced Renal-Cell Carcinoma. Cancer Chemother. Pharmacol. 1998, 42, 415–417. 10.1007/s002800050838. 9771957

[ref22] Kröger N.; Kleeberg U. R.; Mross K.; Edler L.; Hossfeld D. K. Phase II Clinical Trial of Titanocene Dichloride in Patients with Metastatic Breast Cancer. Oncol. Res. Treat. 2000, 23, 60–62. 10.1159/000027075.

[ref23] Caruso F.; Massa L.; Gindulyte A.; Pettinari C.; Marchetti F.; Pettinari R.; Ricciutelli M.; Costamagna J.; Canales J. C.; Tanski J.; Rossi M. (4-Acyl-5-pyrazolonato) Titanium Derivatives: Oligomerization, Hydrolysis, Voltammetry, and DFT Study. Eur. J. Inorg. Chem. 2003, 17, 3221–3232. 10.1002/ejic.200300135.

[ref24] Shavit M.; Peri D.; Manna C. M.; Alexander J. S.; Tshuva E. Y. Active Cytotoxic Reagents Based on Non-Metallocene Non-Diketonato Well-Defined C 2-Symmetrical Titanium Complexes of Tetradentate Bis (Phenolato) Ligands. J. Am. Chem. Soc. 2007, 129, 12098–12099. 10.1021/ja0753086. 17877357

[ref25] Tshuva E. Y.; Peri D. Modern Cytotoxic Titanium (IV) Complexes; Insights on the Enigmatic Involvement of Hydrolysis. Coord. Chem. Rev. 2009, 253, 2098–2115. 10.1016/j.ccr.2008.11.015.

[ref26] Peri D.; Meker S.; Shavit M.; Tshuva E. Y. Synthesis, Characterization, Cytotoxicity, and Hydrolytic Behavior of C2-and C1-Symmetrical TiIV Complexes of Tetradentate Diamine Bis (Phenolato) Ligands: A New Class of Antitumor Agents. Chem.—Eur. J. 2009, 15, 2403–2415. 10.1002/chem.200801310. 19156656

[ref27] Peri D.; Meker S.; Manna C. M.; Tshuva E. Y. Different Ortho and Para Electronic Effects on Hydrolysis and Cytotoxicity of Diamino Bis (Phenolato)“Salan” Ti (IV) Complexes. Inorg. Chem. 2011, 50, 1030–1038. 10.1021/ic101693v. 21214265

[ref28] Immel T. A.; Groth U.; Huhn T.; Öhlschläger P. Titanium Salan Complexes Displays Strong Antitumor Properties in Vitro and in Vivo in Mice. PLoS One 2011, 6, e17869 10.1371/journal.pone.0017869. 21445304 PMC3061874

[ref29] Immel T. A.; Groth U.; Huhn T. Cytotoxic Titanium Salan Complexes: Surprising Interaction of Salan and Alkoxy Ligands. Chem.—Eur. J. 2010, 16, 2775–2789. 10.1002/chem.200902312. 20104550

[ref30] Meker S.; Manna C. M.; Peri D.; Tshuva E. Y. Major Impact of N-Methylation on Cytotoxicity and Hydrolysis of Salan Ti (IV) Complexes: Sterics and Electronics Are Intertwined. Dalton Trans. 2011, 40, 9802–9809. 10.1039/c1dt11108f. 21874187

[ref31] Manna C. M.; Armony G.; Tshuva E. Y. Unexpected Influence of Stereochemistry on the Cytotoxicity of Highly Efficient TiIV Salan Complexes: New Mechanistic Insights. Chem.—Eur. J. 2011, 17, 14094–14103. 10.1002/chem.201102017. 22076809

[ref32] Peri D.; Manna C. M.; Shavit M.; Tshuva E. Y. TiIV Complexes of Branched Diamine Bis (Phenolato) Ligands: Hydrolysis and Cytotoxicity. Eur. J. Inorg. Chem. 2011, 31, 4896–4900. 10.1002/ejic.201100725.

[ref33] Tshuva E. Y.; Goldberg I.; Kol M.; Goldschmidt Z. Coordination Chemistry of Amine Bis (Phenolate) Titanium Complexes: Tuning Complex Type and Structure by Ligand Modification. Inorg. Chem. 2001, 40, 4263–4270. 10.1021/ic010210s. 11487331

[ref34] Tshuva E. Y.; Versano M.; Goldberg I.; Kol M.; Weitman H.; Goldschmidt Z. Titanium Complexes of Chelating Dianionic Amine Bis (Phenolate) Ligands: An Extra Donor Makes a Big Difference. Inorg. Chem. Commun. 1999, 2, 371–373. 10.1016/s1387-7003(99)00096-9.

[ref35] Barroso S.; Coelho A. M.; Gómez-Ruiz S.; Calhorda M. J.; Žižak Ž.; Kalud̵erović G. N.; Martins A. M. Correction: Synthesis, Cytotoxic and Hydrolytic Studies of Titanium Complexes Anchored by a Tripodal Diamine Bis (Phenolate) Ligand. Dalton Trans. 2015, 44, 2497 10.1039/c4dt90194k. 25526919

[ref36] Manne R.; Miller M.; Duthie A.; Guedes da Silva M. F. C. G.; Tshuva E. Y.; Basu Baul T. S. B. Cytotoxic Homoleptic Ti (Iv) Compounds of ONO-Type Ligands: Synthesis, Structures and Anti-Cancer Activity. Dalton Trans. 2019, 48, 304–314. 10.1039/c8dt03747g. 30516219

[ref37] Shpilt Z.; Manne R.; Rohman M. A.; Mitra S.; Tiekink E. R. T.; Basu Baul T. S.; Tshuva E. Y. Homoleptic Ti [ONO] 2 Type Complexes of Amino-acid-tethered Phenolato Schiff-base Ligands: Synthesis, Characterization, Time-resolved Fluorescence Spectroscopy, and Cytotoxicity against Ovarian and Colon Cancer Cells. Appl. Organomet. Chem. 2020, 34, e5309 10.1002/aoc.5309.

[ref38] Burke W. J.; Glennie E. L. M.; Weatherbee C. Condensation of Halophenols with Formaldehyde and Primary Amines1. J. Org. Chem. 1964, 29, 909–912. 10.1021/jo01027a038.

[ref39] Dolomanov O. V.; Bourhis L. J.; Gildea R. J.; Howard J. A. K.; Puschmann H. OLEX2: A Complete Structure Solution, Refinement and Analysis Program. J. Appl. Crystallogr. 2009, 42, 339–341. 10.1107/s0021889808042726.

[ref40] Sheldrick G. M. SHELXT–Integrated Space-Group and Crystal-Structure Determination. Acta Crystallogr., Sect. A: Found. Adv. 2015, 71, 3–8. 10.1107/s2053273314026370. 25537383 PMC4283466

[ref41] Sheldrick G. Crystal Structure Refinement with SHELXL. Acta Crystallogr., Sect. C: Struct. Chem. 2015, 71, 3–8. 10.1107/s2053229614024218. 25567568 PMC4294323

[ref42] Adamo C.; Barone V. Toward Reliable Density Functional Methods without Adjustable Parameters: The PBE0 Model. J. Chem. Phys. 1999, 110, 6158–6170. 10.1063/1.478522.

[ref43] Grimme S.; Antony J.; Ehrlich S.; Krieg H. A Consistent and Accurate Ab Initio Parametrization of Density Functional Dispersion Correction (DFT-D) for the 94 Elements H-Pu. J. Chem. Phys. 2010, 132, 154104 10.1063/1.3382344. 20423165

[ref44] Cossi M.; Barone V.; Cammi R.; Tomasi J. Ab Initio Study of Solvated Molecules: A New Implementation of the Polarizable Continuum Model. Chem. Phys. Lett. 1996, 255, 327–335. 10.1016/0009-2614(96)00349-1.

[ref45] Frisch M. J.; Trucks G. W.; Schlegel H. B.; Scuseria G. E.; Robb M. A.; Cheeseman J. R.; Scalmani G.; Barone V.; Petersson G. A.; Nakatsuji H.; Li X.; Caricato M.; Marenich A. V.; Bloino J.; Janesko B. G.; Gomperts R.; Mennucci B.; Hratchian H. P.; Ortiz J. V.; Izmaylov A. F.; Sonnenberg J. L.; Williams-Young D.; Ding F.; Lipparini F.; Egidi F.; Goings J.; Peng B.; Petrone A.; Henderson T.; Ranasinghe D.; Zakrzewski V. G.; Gao J.; Rega N.; Zheng G.; Liang W.; Hada M.; Ehara M.; Toyota K.; Fukuda R.; Hasegawa J.; Ishida M.; Nakajima T.; Honda Y.; Kitao O.; Nakai H.; Vreven T.; Throssell K.; Montgomery J. A. Jr.; Peralta J. E.; Ogliaro F.; Bearpark M. J.; Heyd J. J.; Brothers E. N.; Kudin K. N.; Staroverov V. N.; Keith T. A.; Kobayashi R.; Normand J.; Raghavachari K.; Rendell A. P.; Burant J. C.; Iyengar S. S.; Tomasi J.; Cossi M.; Millam J. M.; Klene M.; Adamo C.; Cammi R.; Ochterski J. W.; Martin R. L.; Morokuma K.; Farkas O.; Foresman J. B.; Fox D. J. Gaussian 16, Revision B.01; Gaussian, Inc.: Wallingford CT, 2016.

[ref46] Mitin A. V.; Baker J.; Pulay P. An Improved 6-31 G* Basis Set for First-Row Transition Metals. J. Chem. Phys. 2003, 118, 7775–7782. 10.1063/1.1563619.

[ref47] Ditchfield R.; Hehre W. J.; Pople J. A. Self-consistent Molecular-orbital Methods. IX. An Extended Gaussian-type Basis for Molecular-orbital Studies of Organic Molecules. J. Chem. Phys. 1971, 54, 724–728. 10.1063/1.1674902.

[ref48] Hariharan P. C.; Pople J. A. The Influence of Polarization Functions on Molecular Orbital Hydrogenation Energies. Theor. Chim. Acta 1973, 28, 213–222. 10.1007/bf00533485.

[ref49] Hehre W. J.; Ditchfield R.; Pople J. A. Self—Consistent Molecular Orbital Methods. XII. Further Extensions of Gaussian—Type Basis Sets for Use in Molecular Orbital Studies of Organic Molecules. J. Chem. Phys. 1972, 56, 2257–2261. 10.1063/1.1677527.

[ref50] Ansbacher T.; Srivastava H. K.; Martin J. M. L.; Shurki A. Can DFT Methods Correctly and Efficiently Predict the Coordination Number of Copper (I) Complexes? A Case Study. J. Comput. Chem. 2010, 31, 75–83. 10.1002/jcc.21277. 19412907

[ref51] Meker S.; Braitbard O.; Hall M. D.; Hochman J.; Tshuva E. Y. Specific Design of Titanium (IV) Phenolato Chelates Yields Stable and Accessible, Effective and Selective Anticancer Agents. Chem.—Eur. J. 2016, 22, 9986–9995. 10.1002/chem.201601389. 27320784

[ref52] Ganot N.; Meker S.; Reytman L.; Tzubery A.; Tshuva E. Y. Anticancer Metal Complexes: Synthesis and Cytotoxicity Evaluation by the MTT Assay. J. Visualized Exp. 2013, 81, e50767 10.3791/50767. PMC3989495 24300943

[ref53] Nahari G.; Hoffman R. E.; Tshuva E. Y. From Medium to Endoplasmic Reticulum: Tracing Anticancer Phenolato Titanium (IV) Complex by 19F NMR Detection. J. Inorg. Biochem. 2021, 221, 111492 10.1016/j.jinorgbio.2021.111492. 34051630

[ref54] Meker S.; Margulis-Goshen K.; Weiss E.; Magdassi S.; Tshuva E. Y. High Antitumor Activity of Highly Resistant Salan–Titanium (IV) Complexes in Nanoparticles: An Identified Active Species. Angew. Chem., Int. Ed. 2012, 51, 10515–10517. 10.1002/anie.201205973. 22961758

[ref55] Manna C. M.; Armony G.; Tshuva E. Y. New Insights on the Active Species and Mechanism of Cytotoxicity of Salan-Ti (IV) Complexes: A Stereochemical Study. Inorg. Chem. 2011, 50, 10284–10291. 10.1021/ic201340m. 21923127

