# Supplementary material for: Hydrolytically Stable and Cytotoxic [ONON]2Ti(IV)-Type Octahedral Complexes
Source: Inorg Chem. 2022 Oct 23;61(44):17653–61. doi: 10.1021/acs.inorgchem.2c02737 (PMC9644366; doi:10.1021/acs.inorgchem.2c02737)
Supplement: Supplementary file 1 — ic2c02737_si_001.pdf [file ic2c02737_si_001.pdf]

Supporting Information

Hydrolytically Stable and Cytotoxic [ONO*N*]$_2$Ti(IV) type octahedral complexes

Anastasia Pedko,[a] Eden Rubanovich[b], Edit Y. Tshuva[a,]*, Avital Shurki[b,*]

[a] Institute of Chemistry, Edmond J Safra Campus, The Hebrew University of Jerusalem, Jerusalem 9190401, Israel. Edit.tshuva@mail.huji.ac.il

[b] Institute for drug research, School of pharmacy, Ein Kerem Campus, The Hebrew University of Jerusalem, Jerusalem, 9112001, Israel. Avital.shurki@mail.huji.ac.il

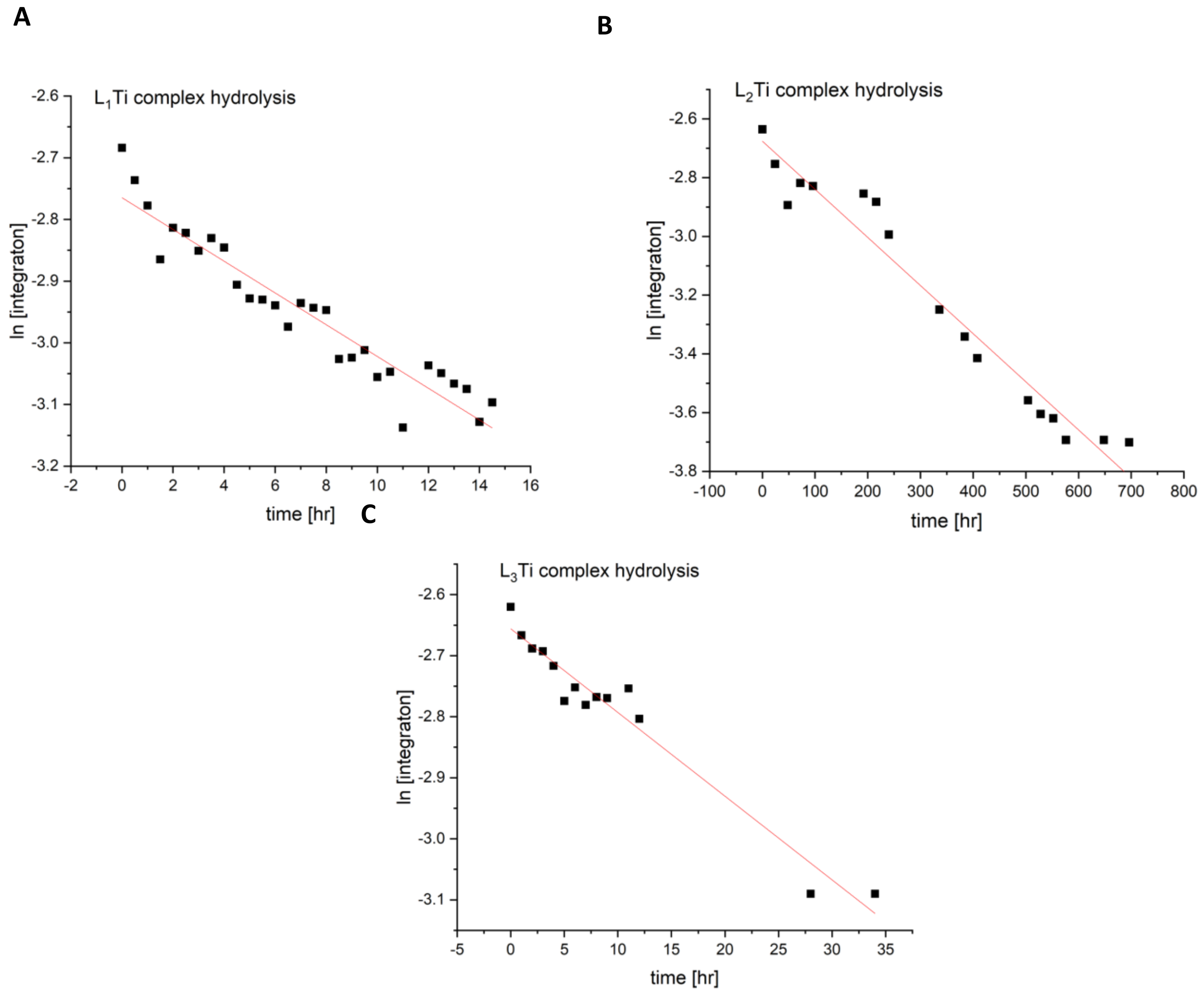

Figure S1. Representative hydrolysis plots of $L^1{}_2Ti$ (A), $L^2{}_2Ti$ (B), and $L^3{}_2Ti$ (C) considering decay of peaks of the complex in the aromatic region

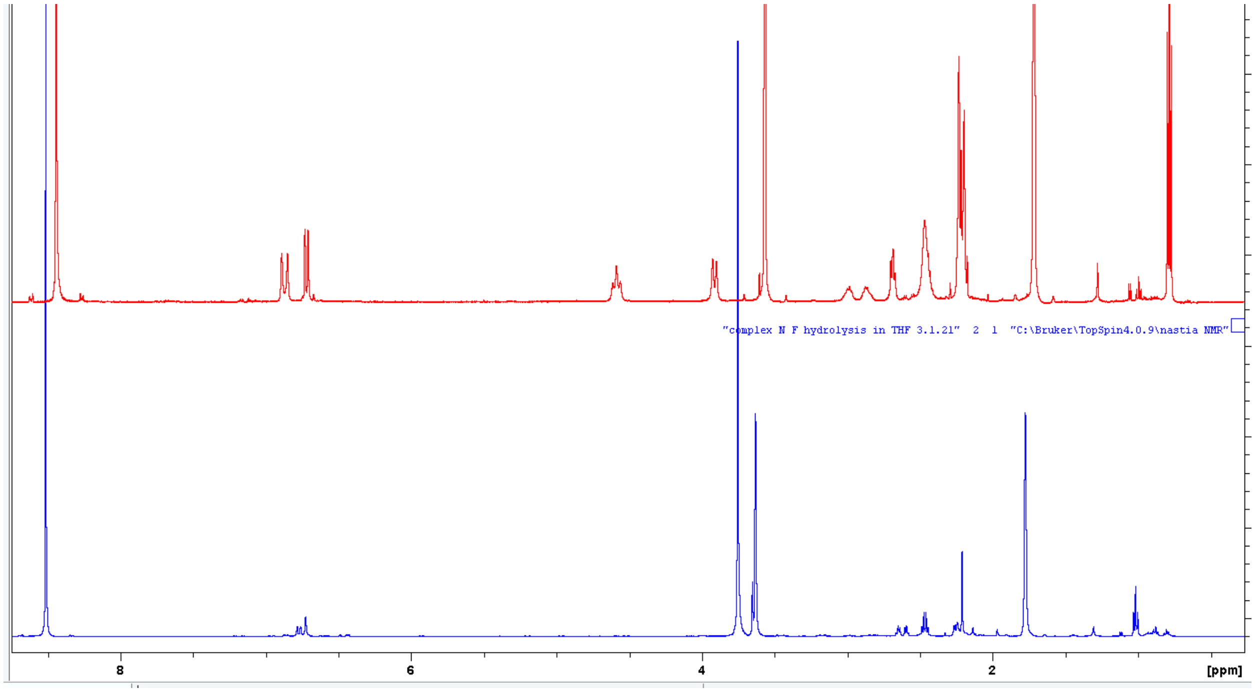

Figure S2. ^{1}H NMR spectra of $L^4{}_2Ti$ in THF-d_8 before (top) and immediately after (bottom) the addition of 10% D_2O.

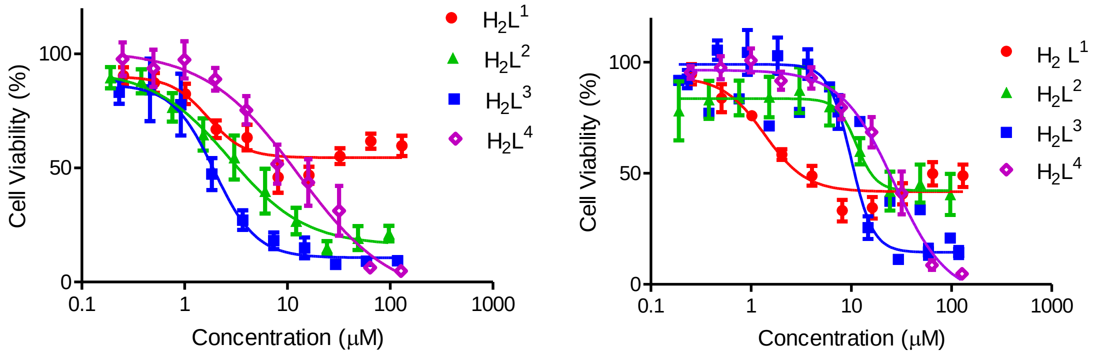

Figure S3. Dependence of human ovarian A2780 (left) and colon HT-29 (right) cancer cell viability on different concentrations of $H_2L^{1\text{-}4}$ following a three-day incubation period as analyzed by the MTT assay

Table S1. IC_{50} (μM) and maximal inhibition (MI; %) values of $H_2L^{1\text{-}4}$ and $L^{1\text{-}4}{}_2Ti$ toward human ovarian A2780 and colon HT-29 cancer cell lines

	**Ligand H^2L**				**Complex L_2Ti**			
	A-2780		HT-29		A-2780		HT-29	
	IC_{50} (μM)	MI (%)	IC_{50} (μM)	MI (%)	IC_{50} (μM)	MI (%)	IC_{50} (μM)	MI (%)
L^1	1.9 ± 0.7	35.6 ± 5.2	1.3 ± 0.4	51.7 ± 6.9	1.6 ± 0.4	91.9 ± 4.7	1.1 ± 0.2	98.2 ± 7.7
L^2	1.5 ± 0.1	76.8 ± 4.0	10 ± 3	41.4 ± 6.9	1.5 ± 0.6	79.6 ± 2.5	13.0 ± 5.7	84.3 ± 18
L^3	1.4 ± 0.6	76.1 ± 8.1	10 ± 3	84.6 ± 4.5	0.6 ± 0.3	88.8 ± 5.5	4.1 ± 1.6	79.0 ± 5.6
L^4	7.4 ± 1.5	98.2 ± 10.6	26.9 ± 6.2	84.7 ± 10.9	6.4 ± 0.7	85.5 ± 4.5	19.9 ± 1.7	96.2 ± 13

Computational Details: All calculations were were performed with Gaussian 16 program package and used the following keywords in the input files: PBE1PBE/gen, EmpiricalDispersion=GD3, freq, and opt. Calculations using the SCRF solvent model involved the additional keyword SCRF=(Solvent=X), where X is either Acetonitrile or n-Pentane using the crystal structures whenever possible as the initial geometry. The optimized geometry of the bound titanium complexes were than reoptimized using the keyword opt=calcfc instead of opt. For all calculations the Pulay's m6-31G* basis set was used for the Titanium atom and the 6-31G* basis set for the rest of the atoms. Optimized geometries of all structures are given below (Tables 2-15). The absolute energies of all compounds are given in Table 16.

Table S2. Optimized geometry of $L^5Ti(OiPr)_2$ calculated using PBE0-D3 with the m6-31G* basis set for Ti and 6-31G* for H, C, N and O in Acetonitrile. All entries are in Å.

Atomic number	Symbol	X	Y	Z
6	C	5.367951	-3.772398	-0.110621
6	C	4.417154	-2.608469	-0.093571
6	C	3.160007	-2.722731	0.499319
6	C	6.110175	-1.205092	-1.34002
6	C	4.769919	-1.377333	-0.681808
6	C	2.249583	-1.66682	0.541411
6	C	0.931809	-1.801564	1.237541
6	C	3.861239	-0.322108	-0.656613
6	C	2.601869	-0.443685	-0.050714
6	C	-0.319315	-2.027152	-0.837183
6	C	0.978554	0.910209	4.188711
6	C	-1.420884	-1.587829	1.323912
6	C	-3.803829	-2.143086	0.695888
6	C	1.170748	1.536956	2.81421
6	C	-2.751283	-1.231951	0.740125
6	C	-0.993973	-1.200607	-1.916904
6	C	-6.175877	-2.820847	0.179897
6	C	0.853595	0.0985	-2.786403
6	C	-5.067528	-1.805904	0.207209
6	C	1.281178	3.052794	2.90274
6	C	-2.958689	0.071042	0.258153
6	C	-5.276363	-0.494088	-0.260878
6	C	-4.22396	0.420481	-0.231953
6	C	-1.34237	1.019465	-2.746125
6	C	2.235264	3.618269	-1.060209
6	C	-6.619027	-0.083059	-0.797157
6	C	0.723391	3.674527	-1.224544
6	C	0.143988	5.013954	-0.792279
1	H	6.310295	-3.535122	0.400319
1	H	4.931705	-4.645746	0.383855
1	H	5.633488	-4.065862	-1.134701
1	H	6.246976	-1.918282	-2.162933
1	H	2.875188	-3.672271	0.951356
1	H	6.929716	-1.383809	-0.632327
1	H	6.228445	-0.195939	-1.745292
1	H	0.772391	-2.855844	1.510982
1	H	0.929988	-1.218889	2.16441

1	H	-1.419484	-2.65294	1.601715
1	H	0.898865	-0.179789	4.116897
1	H	0.704681	-2.262057	-1.140189
1	H	-0.848345	-2.983797	-0.722348
1	H	1.820491	1.150389	4.847839
1	H	4.116722	0.630262	-1.115668
1	H	-3.632358	-3.155509	1.060031
1	H	-1.248013	-1.004191	2.236132
1	H	2.095256	1.140856	2.36292
1	H	0.058247	1.289256	4.6488
1	H	-6.548693	-2.989077	-0.838971
1	H	1.549964	-0.607905	-2.334759
1	H	-5.838414	-3.784207	0.574403
1	H	-0.921413	-1.733939	-2.87794
1	H	2.155051	3.351264	3.492847
1	H	-2.054432	-1.082244	-1.693053
1	H	0.666817	-0.199216	-3.829258
1	H	-7.037943	-2.498348	0.778445
1	H	1.319784	1.086145	-2.770834
1	H	1.36899	3.481323	1.900336
1	H	0.383295	3.469164	3.37532
1	H	2.608145	2.623614	-1.316255
1	H	2.517312	3.82663	-0.022071
1	H	-1.562626	0.620335	-3.747599
1	H	-6.914153	-0.701212	-1.654666
1	H	-2.268536	1.105923	-2.176871
1	H	-0.90261	2.012791	-2.848423
1	H	2.715191	4.361888	-1.705946
1	H	-4.368309	1.433404	-0.601735
1	H	-6.616622	0.9621	-1.119893
1	H	-7.405575	-0.201959	-0.041066
1	H	0.488247	3.524607	-2.291337
1	H	0.360004	5.192431	0.267449
1	H	-0.94226	5.023221	-0.929635
1	H	0.577482	5.831888	-1.378315
7	N	-0.241165	-1.319372	0.458724
7	N	-0.403671	0.14511	-2.030179
8	O	1.759686	0.581089	-0.061053
8	O	0.085824	1.185346	1.989473
8	O	-1.961295	0.945909	0.232371
8	O	0.095112	2.65229	-0.498223
22	Ti	-0.066986	1.004754	0.174767

Table S3. Optimized geometry of $L^5{}_2Ti$ calculated using PBE0-D3 with the m6-31G* basis set for Ti and 6-31G* for H, C, N and O in Acetonitrile. All entries are in Å.

Atomic cnumber	Symbol	X	Y	Z
6	C	-2.25674	5.077583	-3.600722
6	C	-1.474832	3.953972	-2.979675
6	C	-0.297452	4.213101	-2.248092
6	C	-1.905728	2.632142	-3.107251
6	C	0.40629	3.155627	-1.672944
6	C	-1.208799	1.572508	-2.531502
6	C	-1.638393	0.14361	-2.618052
6	C	-0.045602	1.83951	-1.796885
6	C	5.12849	2.156068	0.149746
6	C	3.536338	-4.021739	-2.089771
6	C	2.38124	-3.24155	-2.099294
6	C	5.673791	0.02484	-0.818287
6	C	5.533781	-4.97397	-0.856956

6	C	4.276162	-4.152068	-0.897445
6	C	1.934422	-2.588621	-0.945457
6	C	-2.334124	-1.860649	-1.479344
6	C	-4.322399	0.428924	-1.182174
6	C	2.935413	0.129492	0.535392
6	C	-2.934128	0.350045	-0.53942
6	C	4.316807	0.119463	1.19687
6	C	3.82001	-3.492952	0.245123
6	C	2.659373	-2.722264	0.248678
6	C	-4.993501	2.498819	-0.101882
6	C	-2.866205	-2.514804	-0.240937
6	C	-0.116678	3.224197	1.617225
6	C	-4.067209	-3.221342	-0.238748
6	C	0.663642	4.207208	2.224514
6	C	0.205477	1.870437	1.74339
6	C	2.168504	-2.02623	1.48059
6	C	1.78735	3.833863	2.99036
6	C	1.313622	1.490321	2.512424
6	C	2.650804	4.87476	3.646515
6	C	-2.142533	-2.411296	0.957325
6	C	-5.658998	0.393788	0.848471
6	C	2.087811	2.476592	3.119103
6	C	1.606448	0.027446	2.603085
6	C	-4.566132	-3.845925	0.905198
6	C	-5.865717	-4.599549	0.86174
6	C	-2.633171	-3.029219	2.112972
6	C	-3.8284	-3.745981	2.101731
1	H	-1.654176	5.643008	-4.323511
1	H	-3.140073	4.700574	-4.125301
1	H	-2.598457	5.798747	-2.84677
1	H	-2.813598	2.422544	-3.671806
1	H	6.042903	2.608895	-0.251156
1	H	-0.842317	-0.473135	-3.052787
1	H	-2.524047	0.041261	-3.258096
1	H	4.315262	2.360371	-0.571584
1	H	4.89257	0.088296	-1.598411
1	H	6.28515	-4.600159	-1.564626
1	H	5.344014	-6.021165	-1.126178
1	H	-4.264035	0.958062	-2.141303
1	H	-3.08863	-1.910878	-2.275226
1	H	5.833638	-1.034716	-0.58955
1	H	2.996378	-0.315228	-0.459333
1	H	-1.442022	-2.382789	-1.846271
1	H	-4.701966	-0.578611	-1.389028
1	H	2.589569	1.15564	0.400254
1	H	-4.726439	2.999726	-1.039437
1	H	-2.515443	1.350644	-0.419504
1	H	5.981125	-4.961822	0.141588
1	H	-4.635185	-3.287983	-1.166051
1	H	4.632006	-0.910883	1.399647
1	H	4.276293	0.643204	2.159734
1	H	4.389542	-3.584171	1.16933
1	H	-3.012469	-0.079589	0.460483
1	H	3.083475	5.565004	2.910552
1	H	-4.159358	2.643363	0.609885
1	H	-6.304904	-4.571252	-0.140088
1	H	2.911955	-2.125852	2.282182
1	H	2.080719	5.490067	4.354742
1	H	1.238067	-2.480102	1.843213
1	H	-5.888518	-0.65054	0.609309
1	H	2.953175	2.179084	3.710245
1	H	3.477014	4.412809	4.195674
1	H	2.466459	-0.155524	3.260017

1	H	-4.865863	0.39748	1.619076
1	H	0.749577	-0.514484	3.021947
1	H	-5.734014	-5.653322	1.139893
1	H	-6.600824	-4.180806	1.561273
7	N	5.358148	0.74668	0.398472
7	N	-1.918346	-0.442816	-1.274516
7	N	-5.313409	1.109585	-0.363688
7	N	1.857691	-0.583081	1.264537
8	O	0.599084	0.812552	-1.240125
8	O	0.850998	-1.818132	-0.975837
8	O	-0.514894	0.914016	1.156263
8	O	-1.020458	-1.698454	0.993992
22	Ti	-0.024166	-0.476929	-0.012503
1	H	4.879292	2.661731	1.089638
1	H	6.598408	0.42693	-1.248777
1	H	-5.872538	3.002371	0.317228
1	H	-6.550781	0.848256	1.295775
6	C	3.99368	-4.708607	-3.345809
1	H	3.314864	-4.508441	-4.179782
1	H	4.997748	-4.377212	-3.63963
1	H	4.050697	-5.795946	-3.208858
1	H	1.806964	-3.119562	-3.014723
1	H	-2.059708	-2.930051	3.031698
6	C	-4.330598	-4.396567	3.359934
1	H	-5.316903	-4.008457	3.644354
1	H	-4.446072	-5.480251	3.231085
1	H	-3.647139	-4.22696	4.19688
1	H	-0.973346	3.499514	1.007804
6	C	0.316005	5.658275	2.042207
1	H	0.13833	6.155022	3.004437
1	H	1.132448	6.205023	1.552957
1	H	-0.581661	5.777387	1.428326
1	H	1.303985	3.344192	-1.089926
6	C	0.189463	5.623656	-2.066377
1	H	0.383508	6.111522	-3.029999
1	H	-0.557372	6.238968	-1.548105
1	H	1.112428	5.652706	-1.479732

Table S4. Optimized geometry of L^5H_2 calculated using PBE0-D3 with the 6-31G* basis set for H, C, N and O in Acetonitrile. All entries are in Å.

Atomic number	Symbol	X	Y	Z
6	C	-5.649348	-1.045017	1.35763
6	C	-4.548739	-0.693018	0.39648
6	C	-3.387722	-1.465373	0.325762
6	C	-5.876934	1.302361	-0.401472
6	C	-4.656922	0.426663	-0.447052
6	C	-2.334428	-1.174156	-0.539536
6	C	-1.073894	-1.989236	-0.56509
6	C	-3.6169	0.720717	-1.329802
6	C	-2.467335	-0.065074	-1.382509
6	C	-0.03762	-0.904416	1.361246
6	C	1.335504	-1.923903	-0.380884
6	C	3.725993	-1.421711	0.296661
6	C	2.534037	-1.016864	-0.300762
6	C	0.375418	0.52482	1.700385
6	C	6.139958	-1.114744	0.955471
6	C	-1.739514	1.676086	1.634415
6	C	4.875852	-0.630393	0.301458
6	C	2.48507	0.252431	-0.902068

6	C	4.820556	0.631786	-0.319464
6	C	3.627051	1.056224	-0.902904
6	C	0.259548	2.805515	0.98847
6	C	6.030165	1.524004	-0.339757
1	H	-6.597347	-1.236338	0.838331
1	H	-5.398005	-1.940525	1.934015
1	H	-5.840703	-0.231841	2.069769
1	H	-6.015404	1.739061	0.59579
1	H	-3.290248	-2.330354	0.980776
1	H	-6.78813	0.732566	-0.623083
1	H	-5.80814	2.122798	-1.121617
1	H	-1.224508	-2.923544	0.003626
1	H	-0.845568	-2.27376	-1.598944
1	H	1.489121	-2.813604	0.255549
1	H	-1.077613	-1.056529	1.665102
1	H	0.566685	-1.597036	1.969055
1	H	-3.703074	1.58322	-1.98939
1	H	3.758456	-2.40131	0.772772
1	H	1.243211	-2.287207	-1.414135
1	H	6.00486	-2.112665	1.384092
1	H	-2.290433	0.732721	1.589938
1	H	6.973127	-1.16786	0.24235
1	H	0.341394	0.639236	2.803763
1	H	1.418739	0.671931	1.407095
1	H	-1.688932	2.00491	2.69228
1	H	6.463317	-0.445457	1.763597
1	H	-2.321812	2.415185	1.074315
1	H	0.469456	3.224469	1.99288
1	H	6.370214	1.762113	0.676296
1	H	1.208267	2.689827	0.454622
1	H	-0.350834	3.53529	0.444131
1	H	3.563026	2.036675	-1.369636
1	H	5.822299	2.466465	-0.854851
1	H	6.875675	1.041214	-0.846427
7	N	0.08211	-1.237796	-0.06243
7	N	-0.42975	1.532541	1.032785
8	O	-1.452218	0.185776	-2.250161
8	O	1.35655	0.724466	-1.47435
1	H	0.589778	0.210528	-1.095871
1	H	-1.636818	1.004456	-2.732645

Table S5. Optimized geometry of iPrOH calculated using PBE0-D3 with the 6-31G* basis set for H, C and O in Acetonitrile. All entries are in Å.

Atomic number	Symbol	X	Y	Z
6	C	0.001359	0.045304	0.364133
6	C	-1.179296	-0.79445	-0.102501
6	C	1.328959	-0.532922	-0.088052
8	O	-0.06664	1.364167	-0.166519
1	H	-0.007922	0.08845	1.466399
1	H	-2.128504	-0.352873	0.224992
1	H	-1.188201	-0.855813	-1.196976
1	H	-1.124679	-1.810651	0.30364
1	H	2.157692	0.100003	0.245706
1	H	1.472104	-1.538327	0.320494
1	H	1.360062	-0.594163	-1.182205
1	H	-0.913564	1.742445	0.108629

Table S6. Optimized geometry of $L^5Ti(OiPr)_2$ calculated using PBE0-D3 with the m6-31G* basis set for Ti and 6-31G* for H, C, N and O in n-Pentane. All entries are in Å .

Atomic number	Symbol	X	Y	Z
6	C	5.363516	-3.785399	-0.000805
6	C	4.41408	-2.620747	-0.024047
6	C	3.151794	-2.716773	0.558333
6	C	6.122174	-1.248974	-1.283377
6	C	4.773848	-1.404808	-0.637809
6	C	2.24082	-1.660176	0.56752
6	C	0.923591	-1.786375	1.268554
6	C	3.86635	-0.350186	-0.648445
6	C	2.599559	-0.450451	-0.052399
6	C	-0.35497	-2.065378	-0.785627
6	C	1.46271	1.117408	3.92604
6	C	-1.42945	-1.549276	1.371079
6	C	-3.819393	-2.120834	0.778059
6	C	1.075782	1.902269	2.680918
6	C	-2.760633	-1.216959	0.776085
6	C	-1.003879	-1.249912	-1.892613
6	C	-6.199336	-2.801055	0.307216
6	C	0.898427	-0.068851	-2.792423
6	C	-5.081943	-1.796398	0.279895
6	C	0.515475	3.276482	3.018827
6	C	-2.960107	0.064055	0.235166
6	C	-5.282155	-0.506082	-0.246806
6	C	-4.223858	0.400578	-0.266014
6	C	-1.251854	0.958415	-2.802474
6	C	2.215866	3.81956	-0.390142
6	C	-6.625447	-0.10847	-0.791515
6	C	0.990885	3.487897	-1.229721
6	C	0.216178	4.730441	-1.643961
1	H	6.299651	-3.537785	0.516864
1	H	4.920635	-4.645938	0.510343
1	H	5.641482	-4.106834	-1.013348
1	H	6.271293	-1.982827	-2.085925
1	H	2.862908	-3.654113	1.033541
1	H	6.932885	-1.406199	-0.560441
1	H	6.245055	-0.250653	-1.713442
1	H	0.769901	-2.837552	1.561663
1	H	0.918476	-1.18441	2.182915
1	H	-1.429171	-2.602052	1.69623
1	H	1.91281	0.156587	3.654771
1	H	0.661485	-2.3438	-1.078979
1	H	-0.918339	-2.999218	-0.643645
1	H	2.189847	1.674955	4.527571
1	H	4.124269	0.591957	-1.126998
1	H	-3.655582	-3.116057	1.190731
1	H	-1.251393	-0.924243	2.254552
1	H	1.970422	2.023573	2.050506
1	H	0.575547	0.926257	4.540885
1	H	-5.867642	-3.748809	0.742755
1	H	1.556222	-0.79199	-2.310487
1	H	-7.053065	-2.444993	0.898791

1	H	-0.956624	-1.821064	-2.834951
1	H	1.266868	3.899056	3.518575
1	H	-2.057959	-1.08433	-1.668732
1	H	0.708634	-0.390676	-3.828761
1	H	-6.583019	-3.0137	-0.699428
1	H	1.417736	0.891138	-2.801109
1	H	0.191607	3.777464	2.101385
1	H	-0.353619	3.17629	3.679218
1	H	2.721919	2.895798	-0.098124
1	H	1.919477	4.360335	0.515526
1	H	-1.490506	0.541166	-3.793465
1	H	-6.934637	-0.7625	-1.61709
1	H	-2.17143	1.11235	-2.236426
1	H	-0.761111	1.925601	-2.929435
1	H	2.913633	4.447388	-0.956254
1	H	-4.360667	1.398809	-0.675262
1	H	-6.616087	0.920452	-1.162706
1	H	-7.406409	-0.182058	-0.023883
1	H	1.329637	2.964538	-2.139532
1	H	-0.132042	5.265326	-0.753242
1	H	-0.660817	4.45586	-2.239396
1	H	0.84537	5.405856	-2.234687
7	N	-0.253854	-1.324653	0.487879
7	N	-0.361221	0.063658	-2.054695
8	O	1.766447	0.576041	-0.108729
8	O	0.103591	1.180387	1.958094
8	O	-1.954226	0.922482	0.161116
8	O	0.126648	2.633583	-0.528631
22	Ti	-0.071363	0.993094	0.158398

Table S7. Optimized geometry of $L^5{}_2Ti$ calculated using PBE0-D3 with the m6-31G* basis set for Ti and 6-31G* for H, C, N and O in n-Pentane. All entries are in Å.

Atomic number	Symbol	X	Y	Z
6	C	-2.290722	5.034502	-3.636416
6	C	-1.502369	3.922872	-3.00249
6	C	-0.336221	4.197954	-2.259839
6	C	-1.915271	2.596006	-3.129695
6	C	0.375901	3.152225	-1.675704
6	C	-1.210529	1.547534	-2.544903
6	C	-1.622502	0.113472	-2.62372
6	C	-0.056414	1.829761	-1.801299
6	C	5.110397	2.18939	0.196692
6	C	3.577056	-3.970198	-2.109584
6	C	2.412763	-3.205674	-2.114796
6	C	5.668009	0.077961	-0.805444
6	C	5.581275	-4.913341	-0.881226
6	C	4.31477	-4.105226	-0.917094
6	C	1.952812	-2.571863	-0.955994
6	C	-2.318453	-1.885556	-1.472731
6	C	-4.314814	0.400393	-1.192877
6	C	2.927512	0.139472	0.544705
6	C	-2.927203	0.330692	-0.548854
6	C	4.3078	0.13244	1.207319
6	C	3.846905	-3.464576	0.230383
6	C	2.677221	-2.709083	0.23804
6	C	-4.995895	2.477149	-0.13567
6	C	-2.856651	-2.528599	-0.23186
6	C	-0.122385	3.213106	1.622102

6	C	-4.059933	-3.229706	-0.224509
6	C	0.654535	4.192644	2.23729
6	C	0.195086	1.858428	1.74951
6	C	2.172461	-2.029046	1.472631
6	C	1.77038	3.817292	3.012631
6	C	1.297728	1.476645	2.526001
6	C	2.627764	4.855766	3.680126
6	C	-2.132135	-2.416843	0.965106
6	C	-5.656597	0.37964	0.832875
6	C	2.067882	2.460036	3.14076
6	C	1.589418	0.013477	2.60823
6	C	-4.562429	-3.840883	0.924268
6	C	-5.864109	-4.590937	0.886722
6	C	-2.627743	-3.02057	2.1256
6	C	-3.825327	-3.731451	2.119714
1	H	-1.688084	5.603999	-4.356084
1	H	-3.163467	4.645071	-4.169924
1	H	-2.651609	5.755042	-2.890536
1	H	-2.815244	2.373489	-3.702435
1	H	6.026887	2.653288	-0.187057
1	H	-0.812762	-0.496143	-3.043322
1	H	-2.501647	-0.003579	-3.271732
1	H	4.301882	2.404614	-0.527187
1	H	4.89308	0.155339	-1.590475
1	H	6.332081	-4.525071	-1.581938
1	H	5.40458	-5.960622	-1.159382
1	H	-4.25684	0.921052	-2.157194
1	H	-3.068953	-1.945288	-2.273019
1	H	5.823607	-0.985851	-0.594683
1	H	2.990766	-0.295044	-0.454336
1	H	-1.421323	-2.40855	-1.826365
1	H	-4.692072	-0.610238	-1.389919
1	H	2.575176	1.164471	0.41798
1	H	-4.722317	2.96796	-1.076783
1	H	-2.511603	1.333456	-0.436635
1	H	6.025992	-4.906429	0.11874
1	H	-4.626737	-3.30468	-1.152265
1	H	4.631606	-0.898282	1.395486
1	H	4.261902	0.642921	2.17754
1	H	4.414198	-3.56002	1.15594
1	H	-3.00307	-0.089957	0.455057
1	H	3.069834	5.548561	2.951886
1	H	-4.168408	2.634646	0.581515
1	H	-6.302999	-4.570359	-0.115629
1	H	2.912134	-2.13123	2.278666
1	H	2.052066	5.468689	4.386041
1	H	1.241421	-2.492567	1.821606
1	H	-5.874307	-0.669291	0.603591
1	H	2.927956	2.16033	3.739013
1	H	3.448389	4.392148	4.236556
1	H	2.444027	-0.173819	3.272451
1	H	-4.869958	0.397099	1.609842
1	H	0.726313	-0.53027	3.012067
1	H	-5.736374	-5.643356	1.172235
1	H	-6.60009	-4.164666	1.58105
7	N	5.344416	0.77814	0.42076
7	N	-1.90983	-0.464794	-1.278913
7	N	-5.30796	1.085069	-0.383439
7	N	1.854926	-0.586353	1.268312
8	O	0.591891	0.813533	-1.235496
8	O	0.86127	-1.816297	-0.979156
8	O	-0.517488	0.904855	1.154235
8	O	-1.008376	-1.710274	0.993441

22	Ti	-0.02088	-0.482872	-0.013378
1	H	4.851047	2.676529	1.14365
1	H	6.597714	0.485728	-1.219911
1	H	-5.881563	2.981952	0.268266
1	H	-6.55704	0.830815	1.266487
6	C	4.047384	-4.636789	-3.371748
1	H	3.369005	-4.434806	-4.205683
1	H	5.047897	-4.289565	-3.659787
1	H	4.115828	-5.72524	-3.248632
1	H	1.84046	-3.078018	-3.030239
1	H	-2.055001	-2.912294	3.04332
6	C	-4.331848	-4.367004	3.383868
1	H	-5.316559	-3.971944	3.664686
1	H	-4.45003	-5.451922	3.267497
1	H	-3.648706	-4.191026	4.219814
1	H	-0.972836	3.489441	1.004873
6	C	0.309843	5.644501	2.055253
1	H	0.112061	6.136668	3.01608
1	H	1.13387	6.195637	1.583598
1	H	-0.576422	5.765191	1.42532
1	H	1.264923	3.352556	-1.083819
6	C	0.132055	5.614887	-2.078246
1	H	0.343128	6.097727	-3.040992
1	H	-0.630365	6.227794	-1.579866
1	H	1.042244	5.656699	-1.47273

Table S8. Optimized geometry of L^5H_2 calculated using PBE0-D3 with the 6-31G* basis set for H, C, N and O in n-Pentane. All entries are in Å.

Atomic number	Symbol	X	Y	Z
6	C	-5.67137	-1.124629	1.250645
6	C	-4.544036	-0.721626	0.342224
6	C	-3.389778	-1.501289	0.246725
6	C	-5.831074	1.339155	-0.346862
6	C	-4.619648	0.453919	-0.423921
6	C	-2.314399	-1.165176	-0.571911
6	C	-1.05819	-1.986122	-0.626086
6	C	-3.555733	0.794223	-1.259743
6	C	-2.414926	-0.000275	-1.340054
6	C	-0.008015	-0.957675	1.327368
6	C	1.355376	-1.91492	-0.459104
6	C	3.734792	-1.421775	0.255125
6	C	2.545392	-1.002293	-0.336403
6	C	0.346279	0.485026	1.680056
6	C	6.139127	-1.124672	0.948973
6	C	-1.805014	1.561836	1.727516
6	C	4.877967	-0.624461	0.302008
6	C	2.491579	0.289405	-0.887555
6	C	4.818492	0.660005	-0.269726
6	C	3.628667	1.098649	-0.846838
6	C	0.106403	2.751212	0.933904
6	C	6.021824	1.560338	-0.244583
1	H	-6.607597	-1.27344	0.697041
1	H	-5.442853	-2.058937	1.772484
1	H	-5.874078	-0.359464	2.011378
1	H	-5.989397	1.710088	0.673907
1	H	-3.316718	-2.408824	0.84507
1	H	-6.742857	0.797976	-0.629501
1	H	-5.736773	2.206029	-1.007483

1	H	-1.215961	-2.940148	-0.090735
1	H	-0.83386	-2.236161	-1.669934
1	H	1.521991	-2.834404	0.13235
1	H	-1.034442	-1.162778	1.648902
1	H	0.641614	-1.630709	1.910286
1	H	-3.617043	1.699683	-1.862561
1	H	3.77016	-2.419446	0.69227
1	H	1.261755	-2.226909	-1.508989
1	H	6.007376	-2.139198	1.338172
1	H	-2.316481	0.595141	1.738106
1	H	6.979554	-1.146746	0.242587
1	H	0.341632	0.583448	2.785469
1	H	1.371886	0.684663	1.358329
1	H	-1.706379	1.919094	2.77298
1	H	6.450476	-0.485742	1.78608
1	H	-2.452667	2.258887	1.185886
1	H	0.368875	3.20167	1.912066
1	H	6.351214	1.762891	0.782766
1	H	1.015458	2.656716	0.332724
1	H	-0.569531	3.444435	0.419464
1	H	3.560138	2.094707	-1.277725
1	H	5.81155	2.520371	-0.725131
1	H	6.875089	1.104207	-0.762987
7	N	0.099335	-1.258007	-0.102801
7	N	-0.530305	1.456477	1.051202
8	O	-1.380202	0.286228	-2.170562
8	O	1.367336	0.784366	-1.445294
1	H	0.596979	0.247604	-1.11886
1	H	-1.471681	1.188112	-2.506928

Table S9. Optimized geometry of iPrOH calculated using PBE0-D3 with the 6-31G* basis set for H, C and O in n-Pentane. All entries are in Å.

Atomic number	Symbol	X	Y	Z
6	C	0.001531	0.045761	0.364968
6	C	-1.189986	-0.77922	-0.102552
6	C	1.321462	-0.548363	-0.08862
8	O	-0.046905	1.362423	-0.164778
1	H	-0.005887	0.085737	1.468382
1	H	-2.135151	-0.329643	0.227598
1	H	-1.201417	-0.834042	-1.197034
1	H	-1.148627	-1.798218	0.298419
1	H	2.15493	0.079112	0.242036
1	H	1.456116	-1.55506	0.320172
1	H	1.349226	-0.607594	-1.182539
1	H	-0.891997	1.751252	0.098412

Table S10. Optimized geometry of $L^1Ti(OiPr)_2$ calculated using PBE0-D3 with the m6-31G* basis set for Ti and 6-31G* for H, C, N and O in Acetonitrile. All entries are in Å.

Atomic number	Symbol	X	Y	Z
6	C	-5.667279	-3.697864	0.191027
6	C	1.288062	0.289729	4.334918
6	C	-4.657509	-2.593166	0.343959

6	C	-3.318834	-2.869528	0.629528
6	C	-0.966934	-2.14637	1.167537
6	C	-5.025391	-1.25311	0.190827
6	C	-2.373182	-1.85296	0.741576
6	C	1.472084	0.915162	2.959532
6	C	1.38025	-2.044606	0.956316
6	C	-4.107894	-0.206244	0.281387
6	C	-2.757734	-0.516356	0.538598
6	C	1.751569	2.408545	3.045526
6	C	-4.530678	1.226185	0.131487
6	C	3.697131	-2.523106	0.12288
6	C	2.647933	-1.6151	0.27928
6	C	0.055568	-2.110101	-1.069723
6	C	6.053035	-3.124829	-0.570072
6	C	-1.358874	3.885567	1.54718
6	C	4.936914	-2.134179	-0.381186
6	C	2.827705	-0.277797	-0.11128
6	C	-0.818733	-1.258852	-1.969658
6	C	-1.195868	3.43947	0.101733
6	C	5.10673	-0.786228	-0.717665
6	C	4.0831	0.148945	-0.593848
6	C	-1.546585	0.997236	-2.425967
6	C	-0.901121	4.607874	-0.827073
6	C	4.289914	1.597792	-0.922263
6	C	0.777378	0.339796	-2.820487
6	C	-2.029848	0.755765	-3.850895
6	C	1.187654	1.788294	-3.017113
1	H	-6.652596	-3.394499	0.561471
1	H	-5.362384	-4.597106	0.736573
1	H	1.079772	-0.782422	4.24755
1	H	2.1881	0.418621	4.946675
1	H	0.444697	0.760644	4.85387
1	H	-3.002963	-3.901111	0.779537
1	H	-5.792836	-3.98332	-0.861889
1	H	-0.796755	-1.71296	2.160695
1	H	-0.835433	-3.236169	1.252288
1	H	-6.069077	-1.011359	-0.00727
1	H	1.352816	-1.647811	1.977254
1	H	1.373346	-3.142783	1.036665
1	H	2.329107	0.432437	2.462352
1	H	3.539734	-3.56006	0.416803
1	H	2.670216	2.600039	3.611867
1	H	0.920207	2.921551	3.542332
1	H	-4.14174	1.839588	0.952079
1	H	-5.621301	1.313893	0.117029
1	H	-1.535894	3.017365	2.189138
1	H	5.873528	-4.042412	-0.000494
1	H	-0.308888	-3.147891	-1.079815
1	H	7.015563	-2.71029	-0.249493
1	H	-2.202266	4.578555	1.645895
1	H	1.859827	2.828877	2.041206
1	H	-0.449613	4.388603	1.895154
1	H	-4.147228	1.670716	-0.795582
1	H	-1.857752	-1.316328	-1.642126
1	H	1.078462	-2.127345	-1.456477
1	H	6.162104	-3.40944	-1.624808
1	H	-2.126735	2.947304	-0.217475
1	H	-2.363672	0.823688	-1.723918
1	H	-0.774829	-1.678244	-2.986171
1	H	6.074473	-0.451085	-1.089117
1	H	4.038181	2.232662	-0.063823
1	H	1.600416	-0.204249	-2.352636
1	H	-1.716608	5.339428	-0.802651

1	H	-2.376449	-0.272152	-3.999804
1	H	-1.248417	2.03915	-2.297702
1	H	0.02446	5.109161	-0.519972
1	H	5.328772	1.793476	-1.204363
1	H	-2.875814	1.421168	-4.053825
1	H	-0.775519	4.262847	-1.858894
1	H	1.265093	2.300579	-2.055204
1	H	0.597467	-0.132623	-3.799035
1	H	3.642825	1.92042	-1.744105
1	H	-1.257367	0.96761	-4.597443
1	H	0.48195	2.337752	-3.648664
1	H	2.161606	1.817759	-3.516171
7	N	0.118439	-1.601082	0.313355
7	N	-0.417196	0.159547	-1.964199
8	O	0.313691	0.67441	2.193823
8	O	-1.846385	0.44947	0.579741
8	O	1.833967	0.60191	-0.02315
8	O	-0.152094	2.498272	0.000347
22	Ti	0.01345	0.742556	0.393452

Table S11. Optimized geometry of $L^1{}_2Ti$ calculated using PBE0-D3 with the m6-31G* basis set for Ti and 6-31G* for H, C, N and O in Acetonitrile. All entries are in Å.

Atomic number	Symbol	X	Y	Z
6	C	2.741719	-4.319197	-4.269259
6	C	1.881369	-3.403123	-3.442536
6	C	0.848661	-3.905218	-2.643694
6	C	2.114667	-2.026147	-3.418428
6	C	-1.781379	2.526525	-3.68536
6	C	-7.056508	-0.625927	-1.355646
6	C	0.066807	-3.092138	-1.822078
6	C	1.356952	-1.180848	-2.612737
6	C	-1.043156	-3.639438	-0.975988
6	C	1.577355	0.29768	-2.563324
6	C	0.341997	-1.712982	-1.802548
6	C	-4.909717	-2.455383	-0.070995
6	C	-3.89421	3.370854	-2.563757
6	C	-2.647129	2.754401	-2.482109
6	C	-5.665882	-0.291019	-0.833314
6	C	-4.580823	-3.367382	1.101397
6	C	-6.077927	4.180261	-1.580813
6	C	-4.712492	3.563774	-1.444855
6	C	-2.197435	2.323336	-1.220728
6	C	2.208087	2.24851	-1.319532
6	C	4.305053	0.060534	-1.152007
6	C	-2.911219	-0.306999	0.545592
6	C	2.917247	0.024262	-0.502373
6	C	-4.304658	-0.43577	1.172368
6	C	-4.239535	3.124293	-0.207233
6	C	-2.994305	2.511459	-0.08159
6	C	1.789619	-3.108312	1.531534
6	C	4.909332	-2.283864	-0.920695
6	C	2.663981	2.870925	-0.0367
6	C	0.506335	-2.781128	2.233775
6	C	3.865616	3.566706	0.071014
6	C	6.132958	-3.187207	-0.995268
6	C	-0.190279	-3.724905	2.990718
6	C	-0.038462	-1.490468	2.121263
6	C	-2.478728	2.035515	1.241559
6	C	-1.398347	-3.436596	3.633649
6	C	-1.238682	-1.170385	2.774955
6	C	-2.150018	-4.495172	4.393368
6	C	1.836567	2.738664	1.087526
6	C	5.512731	-0.727879	0.828384
6	C	-1.905827	-2.139733	3.518337
6	C	-1.732519	0.234207	2.637956
6	C	6.012476	0.662108	1.19171
6	C	4.264513	4.140539	1.280574
6	C	5.586746	4.847175	1.402282
6	C	2.210325	3.301701	2.319462
6	C	3.418218	3.99543	2.385284
6	C	1.309749	3.134625	3.507168
1	H	3.079086	-3.830785	-5.189771
1	H	3.640976	-4.62388	-3.716904
1	H	2.205612	-5.232993	-4.545945
1	H	-2.244802	2.929604	-4.590618
1	H	-7.118709	-1.643531	-1.755494
1	H	0.645613	-4.975312	-2.655695
1	H	2.904023	-1.602031	-4.037809
1	H	-1.598598	1.455703	-3.840325

1	H	-7.327402	0.061303	-2.165073
1	H	-0.797658	2.996281	-3.563065
1	H	-5.758491	-2.882053	-0.616804
1	H	-2.012158	-3.225122	-1.281288
1	H	-4.245699	3.70475	-3.539182
1	H	-1.100606	-4.729342	-1.053373
1	H	0.682923	0.83623	-2.898487
1	H	2.399931	0.576891	-3.233996
1	H	-7.797548	-0.528346	-0.555035
1	H	-4.062839	-2.454366	-0.785121
1	H	-4.934449	-0.375576	-1.660204
1	H	-4.466115	-4.397074	0.746495
1	H	-6.829448	3.424838	-1.846909
1	H	-6.097564	4.945083	-2.364627
1	H	4.227025	-0.148496	-2.224748
1	H	-0.90596	-3.372829	0.078419
1	H	2.985318	2.365732	-2.085923
1	H	-5.650228	0.760001	-0.523893
1	H	-5.385982	-3.347954	1.844548
1	H	-3.000811	0.025494	-0.490399
1	H	1.301082	2.740698	-1.691637
1	H	-3.64686	-3.088029	1.601352
1	H	4.731444	1.065343	-1.059329
1	H	1.661154	-3.066279	0.442203
1	H	-2.412979	-1.27888	0.521504
1	H	4.408517	-2.287663	-1.895401
1	H	2.559436	-1.005318	-0.443788
1	H	-6.402671	4.645137	-0.64412
1	H	2.142522	-4.110332	1.793506
1	H	4.504956	3.659652	-0.805975
1	H	-4.706641	0.555338	1.410227
1	H	6.846598	-2.802946	-1.73188
1	H	-4.237964	-0.981377	2.119981
1	H	-4.855542	3.255782	0.681447
1	H	2.972974	0.395994	0.523595
1	H	-2.928662	-4.953297	3.76875
1	H	2.575833	-2.3869	1.783794
1	H	0.224994	-4.728451	3.074785
1	H	4.182276	-2.709918	-0.204438
1	H	5.835943	-4.198969	-1.293998
1	H	5.235773	1.426717	1.084704
1	H	5.86485	5.342446	0.465874
1	H	-3.265856	2.134171	1.999965
1	H	-1.485073	-5.298537	4.726837
1	H	-1.630131	2.645239	1.573927
1	H	4.625875	-0.973238	1.445625
1	H	6.648407	-3.26734	-0.032195
1	H	6.859761	0.944032	0.556269
1	H	-2.837071	-1.878847	4.019666
1	H	-2.649043	-4.078897	5.275286
1	H	-2.651698	0.374955	3.221398
1	H	6.279731	-1.459604	1.10567
1	H	-0.986232	0.942213	3.018241
1	H	5.566692	5.603181	2.194142
1	H	6.345184	0.676082	2.234976
1	H	6.391617	4.140883	1.646432
1	H	1.164412	2.073094	3.742517
1	H	0.313356	3.550514	3.31309
1	H	3.71391	4.433553	3.337653
1	H	1.722135	3.629325	4.391427
7	N	-5.282037	-1.100271	0.320622
7	N	1.856834	0.804769	-1.187286
7	N	5.247763	-0.900159	-0.59592

7	N	-1.97813	0.62889	1.221711
8	O	-0.352055	-0.886171	-1.026189
8	O	-1.025499	1.705601	-1.107664
8	O	0.571031	-0.537722	1.418216
8	O	0.694316	2.066991	0.971188
22	Ti	-0.036858	0.61584	0.051046

Table S12. Optimized geometry of $L^1{}_2H$ calculated using PBE0-D3 with the 6-31G* basis set for H, C, N and O in Acetonitrile. All entries are in Å .

Atomic number	Symbol	X	Y	Z
6	C	5.951492	-2.110183	0.593952
6	C	4.735198	-1.434531	0.021577
6	C	3.461838	-1.980801	0.182812
6	C	1.004155	-2.099268	-0.264947
6	C	4.843421	-0.22906	-0.676172
6	C	2.316706	-1.354351	-0.315956
6	C	-1.384551	-2.194619	-0.089884
6	C	3.724502	0.439982	-1.171985
6	C	2.452919	-0.123001	-0.97559
6	C	3.846827	1.749423	-1.89611
6	C	-3.786032	-1.714511	0.572774
6	C	-2.641079	-1.369767	-0.144385
6	C	-0.175726	-0.946256	1.55512
6	C	-6.199744	-1.359132	1.236431
6	C	-4.977506	-1.001502	0.435006
6	C	-2.678187	-0.261403	-1.005823
6	C	0.702033	0.258758	1.87598
6	C	-4.992742	0.072183	-0.461263
6	C	-3.866979	0.461419	-1.186337
6	C	1.569505	2.396606	1.137622
6	C	-3.899837	1.609157	-2.152715
6	C	-0.848423	1.957941	1.095005
6	C	1.587526	3.16611	2.454002
6	C	-1.116109	3.083307	0.11071
1	H	6.818242	-2.001669	-0.067054
1	H	5.777748	-3.179271	0.753896
1	H	3.352936	-2.932375	0.701515
1	H	6.228968	-1.675723	1.563262
1	H	0.798305	-2.457128	-1.282788
1	H	1.13737	-2.999703	0.361583
1	H	5.827618	0.212352	-0.827459
1	H	-1.243042	-2.682015	-1.063734
1	H	-1.47942	-3.00081	0.657234
1	H	-3.744099	-2.570388	1.245113
1	H	3.40929	1.69277	-2.899125
1	H	4.89457	2.049361	-1.992814
1	H	-6.16946	-2.402537	1.56726
1	H	0.144717	-1.779103	2.206884
1	H	-7.11698	-1.214645	0.655026
1	H	3.308381	2.544454	-1.366847
1	H	1.757449	-0.021209	1.817451
1	H	-1.211554	-0.723966	1.830699
1	H	-6.286577	-0.734664	2.135395
1	H	2.518328	1.863571	1.006418
1	H	0.498014	0.531057	2.925799
1	H	-5.915625	0.634568	-0.597529
1	H	-3.703617	1.27109	-3.177724
1	H	-1.556654	1.150269	0.893691
1	H	1.682471	2.49854	3.316858

1	H	1.496446	3.098469	0.300013
1	H	-4.873979	2.107017	-2.139657
1	H	2.444653	3.847573	2.465107
1	H	-0.886725	2.757427	-0.909207
1	H	-1.040395	2.297177	2.127115
1	H	-3.127631	2.348885	-1.915589
1	H	0.683298	3.769566	2.587441
1	H	-0.535636	3.984707	0.332039
1	H	-2.175742	3.355232	0.150271
7	N	-0.191964	-1.366244	0.154483
7	N	0.501568	1.397333	0.976335
8	O	1.361959	0.559393	-1.431144
8	O	-1.577567	0.120965	-1.699013
1	H	-0.793053	-0.251836	-1.21925
1	H	0.863775	0.853455	-0.594686

Table S13. Optimized geometry of $L^1Ti(OiPr)_2$ calculated using PBE0-D3 with the m6-31G* basis set for Ti and 6-31G* for H, C, N and O in n-Pentane. All entries are in Å.

Atomic number	Symbol	X	Y	Z
6	C	-5.66711	-3.694273	0.224295
6	C	1.310621	0.380469	4.325527
6	C	-4.655429	-2.590218	0.365995
6	C	-3.318002	-2.863634	0.65536
6	C	-0.968047	-2.129997	1.194101
6	C	-5.022678	-1.251513	0.202653
6	C	-2.372855	-1.846309	0.760112
6	C	1.497969	0.961722	2.931669
6	C	1.380516	-2.033334	0.977327
6	C	-4.106409	-0.204519	0.286192
6	C	-2.755775	-0.511523	0.545098
6	C	1.77955	2.456416	2.966616
6	C	-4.527452	1.227557	0.132475
6	C	3.69689	-2.523821	0.148894
6	C	2.646568	-1.61515	0.291001
6	C	0.0533	-2.123276	-1.046022
6	C	6.055425	-3.130861	-0.52867
6	C	-1.390688	3.843366	1.535048
6	C	4.935903	-2.141074	-0.35879
6	C	2.823884	-0.283701	-0.120063
6	C	-0.82209	-1.282693	-1.957322
6	C	-1.207797	3.431441	0.082029
6	C	5.10419	-0.797998	-0.71345
6	C	4.080335	0.137287	-0.605191
6	C	-1.554562	0.968225	-2.433012
6	C	-0.899673	4.618103	-0.818936
6	C	4.284254	1.582378	-0.949745
6	C	0.769263	0.311722	-2.827558
6	C	-2.039519	0.720568	-3.856648
6	C	1.182492	1.759822	-3.022851
1	H	-6.633732	-3.410425	0.655267
1	H	-5.333109	-4.609608	0.724151
1	H	1.105933	-0.694645	4.271597
1	H	2.207006	0.532053	4.937596
1	H	0.461077	0.863215	4.821868
1	H	-3.003245	-3.894009	0.817384
1	H	-5.845428	-3.944485	-0.830034
1	H	-0.801382	-1.673268	2.177541

1	H	-0.835641	-3.218817	1.301954
1	H	-6.066655	-1.011244	0.004013
1	H	1.353414	-1.618223	1.991148
1	H	1.374696	-3.131072	1.077125
1	H	2.353082	0.461882	2.447898
1	H	3.541692	-3.55646	0.459634
1	H	2.703967	2.664731	3.517559
1	H	0.953235	2.984691	3.45517
1	H	-4.122652	1.842359	0.943914
1	H	-5.617997	1.318937	0.134334
1	H	-1.57271	2.959251	2.153648
1	H	5.856078	-4.057821	0.01888
1	H	-0.312957	-3.161509	-1.046719
1	H	7.007444	-2.725764	-0.166575
1	H	-2.237759	4.531464	1.639011
1	H	1.878447	2.842241	1.947463
1	H	-0.487735	4.342107	1.904211
1	H	-4.157068	1.665665	-0.803248
1	H	-1.860766	-1.339078	-1.628066
1	H	1.076891	-2.144354	-1.431665
1	H	6.200283	-3.397138	-1.583887
1	H	-2.135269	2.946383	-0.258876
1	H	-2.369919	0.796017	-1.728565
1	H	-0.777229	-1.716198	-2.969079
1	H	6.072839	-0.466555	-1.086011
1	H	3.983084	2.226633	-0.11538
1	H	1.592286	-0.23255	-2.359726
1	H	-1.711734	5.353505	-0.788733
1	H	-2.381784	-0.309489	-4.001915
1	H	-1.257139	2.011004	-2.307715
1	H	0.025714	5.106211	-0.49242
1	H	5.332448	1.786445	-1.188671
1	H	-2.889607	1.379952	-4.062398
1	H	-0.763171	4.293005	-1.856064
1	H	1.269131	2.269357	-2.060012
1	H	0.587986	-0.161134	-3.806745
1	H	3.671134	1.883747	-1.805088
1	H	-1.269415	0.93363	-4.605514
1	H	0.474367	2.313102	-3.648721
1	H	2.152828	1.787656	-3.529146
7	N	0.117508	-1.602357	0.330479
7	N	-0.423767	0.134776	-1.970982
8	O	0.337879	0.697962	2.176893
8	O	-1.844984	0.452602	0.573293
8	O	1.829623	0.594979	-0.050543
8	O	-0.163297	2.494148	-0.030288
22	Ti	0.014574	0.74923	0.389614

Table S14. Optimized geometry of $L^1{}_2Ti$ calculated using PBE0-D3 with the m6-31G* basis set for Ti and 6-31G* for H, C, N and O in n-Pentane. All entries are in Å.

Atomic number	Symbol	X	Y	Z
6	C	2.708806	-4.302877	-4.301553
6	C	1.857632	-3.385607	-3.467077
6	C	0.825287	-3.886141	-2.6673
6	C	2.097849	-2.010903	-3.436282
6	C	-1.773881	2.50692	-3.689411
6	C	-7.0486	-0.60621	-1.357325
6	C	0.049879	-3.074379	-1.840136

6	C	1.347084	-1.166075	-2.624068
6	C	-1.06126	-3.617886	-0.993916
6	C	1.574246	0.310549	-2.564466
6	C	0.331265	-1.696525	-1.814593
6	C	-4.927644	-2.444752	-0.027271
6	C	-3.889346	3.353935	-2.575912
6	C	-2.639606	2.746297	-2.489078
6	C	-5.64521	-0.292803	-0.856095
6	C	-4.627591	-3.328405	1.174054
6	C	-6.074241	4.166962	-1.601072
6	C	-4.707268	3.555802	-1.458783
6	C	-2.185116	2.331944	-1.224119
6	C	2.218512	2.250365	-1.309436
6	C	4.300986	0.03899	-1.15326
6	C	-2.907364	-0.287411	0.547413
6	C	2.912951	0.014367	-0.504412
6	C	-4.305359	-0.400127	1.16622
6	C	-4.231514	3.130593	-0.218233
6	C	-2.983619	2.525201	-0.08775
6	C	1.764845	-3.114769	1.533563
6	C	4.880595	-2.312121	-0.925755
6	C	2.682652	2.861764	-0.025067
6	C	0.485602	-2.77843	2.238298
6	C	3.891293	3.544193	0.086698
6	C	6.101029	-3.2167	-1.032526
6	C	-0.215727	-3.712792	3.000957
6	C	-0.049024	-1.483944	2.12333
6	C	-2.46347	2.057166	1.235537
6	C	-1.417906	-3.412486	3.648532
6	C	-1.243346	-1.151763	2.781141
6	C	-2.174601	-4.462238	4.414963
6	C	1.859516	2.720935	1.100481
6	C	5.515069	-0.762722	0.817397
6	C	-1.914707	-2.112435	3.531049
6	C	-1.724395	0.256244	2.63847
6	C	6.039863	0.619807	1.172894
6	C	4.300444	4.098737	1.30083
6	C	5.594903	4.856385	1.410658
6	C	2.251945	3.250789	2.341045
6	C	3.465215	3.931992	2.410654
6	C	1.367855	3.047129	3.534591
1	H	2.151084	-5.189861	-4.620264
1	H	3.081685	-3.798541	-5.199545
1	H	3.584897	-4.65446	-3.73968
1	H	-2.243637	2.886482	-4.601705
1	H	-7.142149	-1.635825	-1.718742
1	H	0.616517	-4.955077	-2.684718
1	H	2.887097	-1.587606	-4.05689
1	H	-1.578262	1.435488	-3.821866
1	H	-7.306943	0.057438	-2.190356
1	H	-0.797069	2.993707	-3.577452
1	H	-5.780135	-2.870336	-0.568516
1	H	-2.022483	-3.172259	-1.278043
1	H	-4.244074	3.674515	-3.554667
1	H	-1.144845	-4.703947	-1.097834
1	H	0.680142	0.854407	-2.892047
1	H	2.39722	0.590116	-3.235936
1	H	-7.778968	-0.460995	-0.554526
1	H	-4.07384	-2.481271	-0.732557
1	H	-4.92125	-0.424574	-1.683177
1	H	-4.535949	-4.371265	0.852143
1	H	-6.816851	3.416795	-1.904682
1	H	-6.084758	4.95711	-2.359816

1	H	4.22036	-0.165892	-2.227055
1	H	-0.907171	-3.383009	0.065859
1	H	2.994697	2.368579	-2.078022
1	H	-5.597093	0.767418	-0.583915
1	H	-5.437449	-3.264617	1.909476
1	H	-2.987006	0.03575	-0.492325
1	H	1.311589	2.749785	-1.672478
1	H	-3.688665	-3.057735	1.66939
1	H	4.738352	1.038831	-1.056067
1	H	1.633131	-3.086243	0.444065
1	H	-2.416218	-1.263159	0.535389
1	H	4.356096	-2.310972	-1.888013
1	H	2.545318	-1.012183	-0.452055
1	H	-6.418766	4.602289	-0.657216
1	H	2.118757	-4.113772	1.805977
1	H	4.529294	3.640709	-0.791192
1	H	-4.70491	0.597341	1.382023
1	H	6.799052	-2.825895	-1.780063
1	H	-4.24798	-0.92892	2.124199
1	H	-4.847565	3.268689	0.669786
1	H	2.971054	0.379586	0.523818
1	H	-2.95785	-4.919335	3.795245
1	H	2.550629	-2.389655	1.775951
1	H	0.191604	-4.719384	3.087267
1	H	4.167868	-2.739669	-0.196001
1	H	5.798317	-4.22747	-1.329537
1	H	5.272139	1.39543	1.083664
1	H	6.332458	4.493383	0.686756
1	H	-3.246881	2.164976	1.997814
1	H	-1.513787	-5.267618	4.752019
1	H	-1.608448	2.664387	1.556527
1	H	4.632208	-0.994524	1.44608
1	H	6.637393	-3.301528	-0.081325
1	H	6.879323	0.888393	0.521564
1	H	-2.841776	-1.842164	4.035714
1	H	-2.667384	-4.039195	5.297318
1	H	-2.640933	0.407321	3.224989
1	H	6.275191	-1.505468	1.084973
1	H	-0.968714	0.958257	3.01166
1	H	5.451244	5.928249	1.22056
1	H	6.391282	0.629208	2.210073
1	H	6.030618	4.759696	2.410827
1	H	1.212743	1.977922	3.723874
1	H	0.375592	3.48641	3.372712
1	H	3.777062	4.340044	3.371203
1	H	1.799996	3.498589	4.432607
7	N	-5.279274	-1.074216	0.321473
7	N	1.860705	0.807903	-1.187152
7	N	5.232345	-0.932771	-0.602469
7	N	-1.972207	0.647519	1.222444
8	O	-0.354622	-0.871527	-1.031824
8	O	-1.009803	1.72487	-1.104045
8	O	0.562464	-0.540896	1.412512
8	O	0.712626	2.060397	0.981345
22	Ti	-0.031168	0.622542	0.05225

Table S15. Optimized geometry of L^1H_2 calculated using PBE0-D3 with the m6-31G* basis set for H, C, N and O in n-Pentane. All entries are in Å.

Atomic number	Symbol	X	Y	Z

6	C	5.957174	-2.073818	0.612269
6	C	4.738999	-1.406809	0.034325
6	C	3.469932	-1.96487	0.180249
6	C	1.016637	-2.097313	-0.301841
6	C	4.84329	-0.198795	-0.659269
6	C	2.325294	-1.346443	-0.328539
6	C	-1.374235	-2.189914	-0.152269
6	C	3.724938	0.461554	-1.165165
6	C	2.456201	-0.111414	-0.980811
6	C	3.838944	1.770719	-1.889934
6	C	-3.762307	-1.717989	0.559134
6	C	-2.630024	-1.364404	-0.174219
6	C	-0.174898	-0.983184	1.53112
6	C	-6.164676	-1.372626	1.267652
6	C	-4.954552	-1.003822	0.453653
6	C	-2.680927	-0.244652	-1.018946
6	C	0.693766	0.223195	1.8756
6	C	-4.985392	0.080655	-0.429014
6	C	-3.87477	0.478004	-1.169714
6	C	1.554181	2.368629	1.152838
6	C	-3.924455	1.630953	-2.128298
6	C	-0.862875	1.925675	1.115898
6	C	1.57836	3.130853	2.474049
6	C	-1.136958	3.051719	0.133983
1	H	6.806773	-2.02349	-0.077835
1	H	5.767849	-3.128666	0.836317
1	H	3.364332	-2.920585	0.692375
1	H	6.271936	-1.591276	1.546832
1	H	0.818901	-2.43226	-1.329048
1	H	1.149122	-3.012109	0.30625
1	H	5.825404	0.250504	-0.801032
1	H	-1.231783	-2.638253	-1.144831
1	H	-1.471815	-3.026662	0.562319
1	H	-3.709122	-2.584057	1.217819
1	H	3.398932	1.707618	-2.890949
1	H	4.884599	2.078361	-1.98715
1	H	-6.082541	-2.388747	1.667388
1	H	0.148709	-1.825352	2.171466
1	H	-7.081778	-1.320748	0.669881
1	H	3.292808	2.56211	-1.363096
1	H	1.75108	-0.049714	1.815301
1	H	-1.213549	-0.772107	1.805593
1	H	-6.297834	-0.694656	2.120996
1	H	2.503195	1.838151	1.012468
1	H	0.484559	0.477495	2.930251
1	H	-5.910985	0.643697	-0.54291
1	H	-3.742047	1.296051	-3.156502
1	H	-1.569928	1.117259	0.913188
1	H	1.677984	2.457995	3.332513
1	H	1.475676	3.074585	0.319072
1	H	-4.898901	2.127825	-2.097265
1	H	2.434321	3.813894	2.48787
1	H	-0.909149	2.726807	-0.886469
1	H	-1.055041	2.261956	2.150019
1	H	-3.147481	2.368809	-1.902248
1	H	0.673716	3.731819	2.616525
1	H	-0.559581	3.955471	0.355274
1	H	-2.197625	3.31897	0.176813
7	N	-0.181673	-1.375705	0.125125
7	N	0.487601	1.370657	0.992457
8	O	1.365995	0.563751	-1.444322
8	O	-1.600411	0.154549	-1.729696
1	H	-0.799025	-0.224051	-1.295753

1	H	0.852267	0.83687	-0.618894

Table S16. Absolute energy values for the different studied compounds.

Ligand	Solvent	Parameter	$LTi(OiPr)_2$ energy (au)	LH_2 energy (au)	L_2Ti energy (au)	iPrOH energy (au)
L1	Acetonitrile	E[a]	-2430.19813	-1194.84251	-3236.767129	-194.1263124
		H[b]	-2429.40668	-1194.24244	-3235.609169	-194.010878
		G[c]	-2429.52147	-1194.33359	-3235.768658	-194.044553
L1	n-Pentane	E[a]	-2430.19065	-1194.83552	-3236.756873	-194.1232086
		H[b]	-2429.39852	-1194.23485	-3235.598033	-194.007626
		G[c]	-2429.51345	-1194.32643	-3235.758067	-194.041283
L5	Acetonitrile	E[a]	-2351.6594	-1116.29602	-3079.683199	-194.1263124
		H[b]	-2350.92863	-1115.75687	-3078.646126	-194.010878
		G[c]	-2351.03596	-1115.84111	-3078.791641	-194.044553
L5	n-Pentane	E[a]	-2351.65075	-1116.28882	-3079.671286	-194.1232086
		H[b]	-2350.91932	-1115.7492	-3078.633413	-194.007626
		G[c]	-2351.02662	-1115.83311	-3078.778357	-194.041283

[a] E is the electronic energy [b] H is the sum of electronic and thermal Enthalpies [c] G is the sum of electronic and thermal Free Energies
